# Interpreting the Hall effect of highly doped conducting polymers

**DOI:** 10.1126/sciadv.aej6501

**Published:** 2026-10-21

**Authors:** Yingqiao Ma, Xinglong Ren, Juncheng Fan, Sebastiaan Hoek, Henning Sirringhaus

**Affiliations:** Optoelectronics Group, Cavendish Laboratory, University of Cambridge, JJ Thomson Avenue, Cambridge CB3 0HE, UK.

## Abstract

Accurate determination of carrier density and mobility is fundamental to understanding the transport physics of conducting polymers and optimizing their performance. However, Hall effect measurements, widely used for determining carrier concentrations in other materials, have been of limited use for conducting polymers. Applying the conventional Hall coefficient expression ($R_{H}={\left[n_{H}q\right]}^{-1}$) consistently yields unphysically high carrier densities in heavily doped polymers. Here, we resolve this problem using a quasi–one-dimensional (Q1D) tight-binding model of the band structure of uniaxially aligned polymer films to analyze the p-type to n-type transition in the Hall effect observed upon increased doping. Our Q1D framework achieves excellent agreement between Hall-derived carrier densities and other independent measurements over a wide doping range. Our findings indicate that the transport physics of aligned polymers is governed by the topology of a single Q1D band within a simple band-filling framework and establish Hall measurements as a powerful tool for studying their charge transport physics.

## INTRODUCTION

Hall effect measurements are routinely used to study conducting polymers, but it could be argued that the insights gained from them have been very limited to date. Literature studies (*1*–*3*) show that directly extracting carrier density from the Hall coefficient (*R*_H_) using the relation $R_{H}={\left(n_{H}q\right)}^{-1}$ with elementary charge (*q*) leads to overestimated values of carrier density (*n*_H_) and, consequently, underestimated mobility (μ_H_). In poly(2,5-bis(3-dodecylthiophen-2-yl)thieno[3,2-b]thiophene) (PBTTT), a widely studied model system, discrepancies between carrier density values extracted from Hall effect and other techniques are large: X-ray photoelectron spectroscopy (XPS) and nuclear magnetic resonance measurements indicate that the carrier density cannot be increased beyond one charge per monomer, i.e., ∼9 × 10^20^ cm^−3^ (*4*), even at the highest doping levels. This is limited by the density of sites available for the dopant counterion within the crystalline, lamellar microstructure of PBTTT. In contrast, Hall carrier densities for the same material are frequently reported up to one order of magnitude larger, approaching 10^22^ cm^−3^ (*3*–*5*). These observations are common, and similar unphysically high Hall carrier densities have been reported in other high-performance conducting polymers, such as n-type poly(benzodifurandione) (PBFDO) and poly(2-(2-decyltetradecyl)-5-dodecyl-6-(5-(selenophen-2-yl)thiophen-2-yl)-3-(thiophen-2-yl)-2,5-dihydropyrrolo[3,4-c]pyrrole-1,4-dione) (*6*–*8*). An explanation that has been put forward to explain this behavior is that in these disordered conductors, there is a coexistence of delocalized carriers that contribute to the generation of a Hall voltage and localized carriers, which do not contribute to the generation of the Hall voltage but cause screening and lowering of the observed transverse Hall voltage (*1*, *9*–*11*). While this explanation has successfully explained observations in field-induced Hall effect measurements on molecular crystals (*1*, *12*), its applicability to highly doped polymers with a (near-) metallic temperature dependence of the conductivity is questionable (Supplementary Text).

Here, we study the Hall effect in highly doped, uniaxially aligned PBTTT film as a model system. We report a p-type to n-type transition in both the Hall and Seebeck effects with increasing doping level. Sign changes in thermopower through chemical doping have been observed in some donor-acceptor copolymers (*13*–*15*) but not yet in semicrystalline polythiophene homopolymers such as PBTTT. We demonstrate that the substantial overestimation of Hall carrier density at high doping levels is not a result of Hall voltage screening by localized charge carriers but stems instead from a band-filling effect, which can also explain the observed sign reversal upon doping. Specifically, by interpreting the Hall data within a quasi–one-dimensional (Q1D) electronic structure model, we achieve excellent agreement with carrier densities derived from XPS and gate charging current analysis. Using this methodology, Hall effect measurements are becoming a powerful tool for studying the fundamental charge transport physics of highly doped polymers.

## RESULTS

For our experiments, we use an organic electrochemical transistor (OECT) architecture that allows precisely defining doping levels electrochemically via the applied gate voltage. The device is based on a previously reported design (*16*), featuring a large side gate for ion injection from a solid-state ion-gel electrolyte based on 1-butyl-1-methylpyrrolidinium bis(trifluoromethylsulfonyl)imide/poly(vinylidene fluoride-co-hexafluoropropylene) (BMP TFSI/PVDF-HFP) (Fig. 1A). The device has an integrated on-chip heater and two temperature sensors (*T*_hot_ and *T*_cold_) for the precise generation and calibration of temperature gradients (fig. S1). Following the patterning of the polymer channel into a Hall bar, in situ thermopower, four-point–probe conductivity, and Hall effect measurements were performed using a PPMS DynaCool 14T system. To ensure stable thermoelectric measurements under electrochemical gating, we first established the polymer doping level by applying a gate voltage (*V*_G_) at 300 K. The device was then cooled to 180 K with the gate voltage maintained; this temperature is below the electrolyte’s glass transition temperature (∼200 K), which “freezes” the ions in place and minimizes ionic contributions to the transport signal. Once the ions were immobilized, the *V*_G_ was removed for subsequent Seebeck and Hall effect measurements. The PBTTT films were fabricated by blade coating, followed by thermal annealing at 260°C into the so-called ribbon phase using a previously reported procedure (*17*). This produces polymer films with chain extended, crystalline polymer ribbon domains in which the chains are highly aligned uniaxially along the blade coating direction. Charge transport was investigated with the longitudinal transport direction of the Hall bar either parallel or perpendicular to the polymer chain alignment direction (Fig. 1B). Three different number-average molecular weights (*M*_n_) of PBTTT, *M*_n_ = 22.5, 31.7, and 47.1 kDa (denoted as *M*_1_, *M*_2_ and *M*_3_, respectively; Fig. 1C and fig. S2), were used to probe the influence of the degree of alignment and the length of the crystalline polymer domains on the charge transport properties. Figure 1D illustrates the microstructure of highly doped PBTTT, showing that the TFSI anions reside within the side-chain region with a stoichiometry of approximately one charge per monomer.

**Fig. 1.**
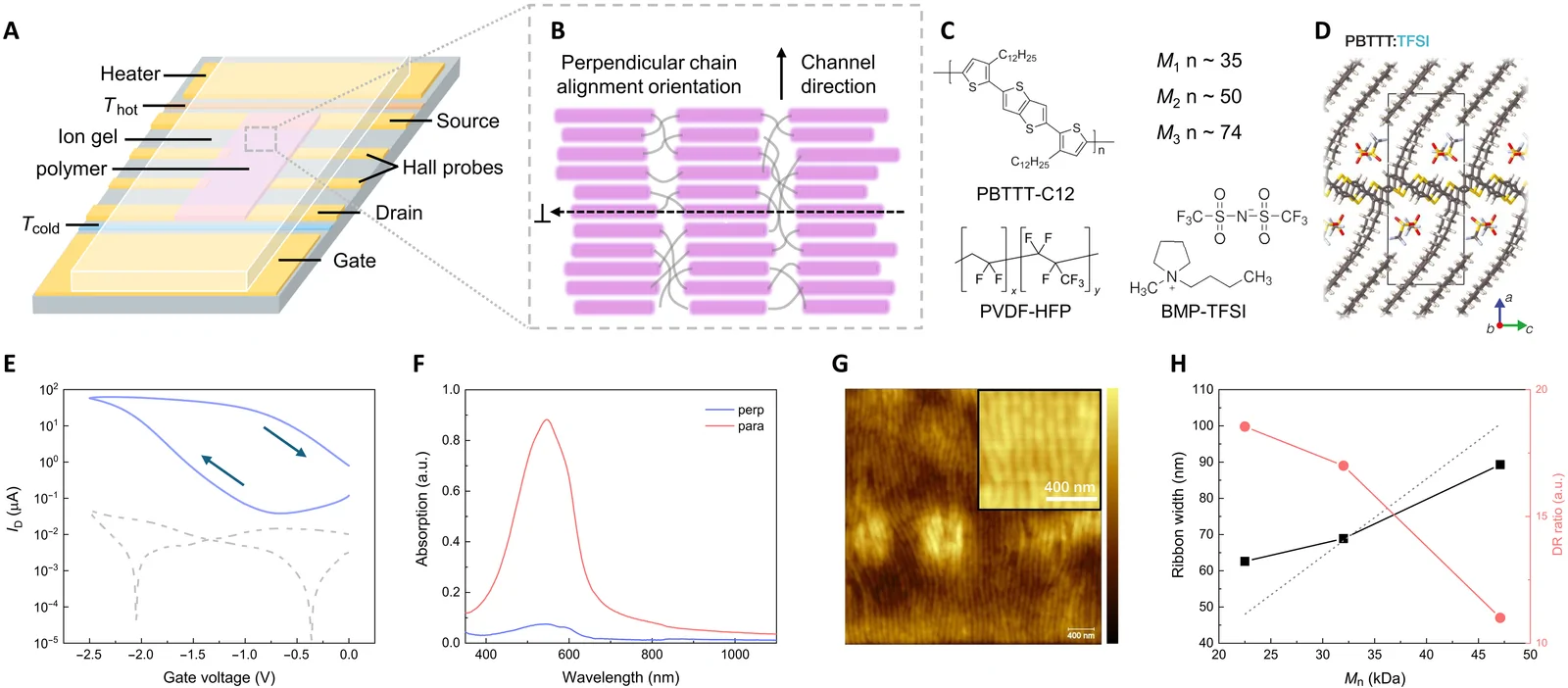
OECTs based on uniaxially aligned, ribbon-phase PBTTT films with different molecular weights. (**A**) Schematic of the side-gate OECT device architecture. All the on-chip electrodes are made of Cr/Au (5/30 nm). A scale diagram and an optical image of the OECT Hall bar device are shown in fig. S10. The detailed device fabrication process can be found in Materials and Methods. (**B**) Schematic illustration of the ribbon-phase PBTTT structure with perpendicular chain alignment orientation with respect to the current flow direction in the channel. (**C**) Chemical structures of PBTTT-C12 (poly(2,5-bis(3-dodecylthiophen-2-yl)thieno[3,2-b]thiophene)) and the ion-gel components (PVDF-HFP and BMP TFSI). The parameter *n* represents the number of monomer repeating units; three molecular weights are used: *M*_1_, *M*_2_, and *M*_3_ (*n* ∼ 35, 50, and 74, respectively). (**D**) Schematic diagram of the TFSI-doped PBTTT lamellar microstructure; figure reproduced from (*4*) under the terms of the Creative Commons CC BY license. (**E**) Typical transfer curve for a parallel-aligned *M*_2_ ribbon-phase PBTTT OECT, recorded at room temperature with a drain voltage *V*_D_ = −0.1 V. The dashed gray line denotes the gate current, and the arrow indicates the voltage sweep direction. (**F**) Polarized ultraviolet–visible–nearinfrared (UV-vis-NIR) spectroscopy of parallel (red) and perpendicular (blue) aligned ribbon-phase PBTTT *M*_2_ films. a.u., arbitrary units; perp, perpendicular; para, parallel. (**G**) Large-area atomic force microscopy (AFM) image of an aligned ribbon-phase PBTTT *M*_2_ film, with a zoom-in inset (top-right) highlighting the uniform chain orientation. (**H**) Correlation plot of the measured ribbon width (from AFM) and dichroic (DR) ratios (from polarized UV-vis) versus molecular weight for PBTTT *M*_1_, *M*_2_, and *M*_3_. The dashed gray line represents the theoretically fully extended chain length calculated on the basis of *n*.

Before low-temperature transport measurements, device functionality was verified at room temperature: Typical OECT transfer curves for a parallel-aligned *M*_2_ device show a well-defined onset near a gate voltage *V*_G_ = −1.0 V with negligible gate leakage (Fig. 1E). The large hysteresis is due to the relatively fast voltage sweep rate. Successful chain alignment was confirmed via polarized ultraviolet–visible–near infrared (UV-vis-NIR) spectroscopy, which yielded a high dichroic (DR) ratio (Fig. 1F and fig. S3). The atomic force microscopy (AFM) images in Fig. 1G and fig. S4 resolve the highly aligned PBTTT nanoribbons. Quantitatively, the ribbon widths measured from AFM images and DR ratios extracted from UV-vis-NIR spectra are plotted versus *M*_n_, verifying that the PBTTT ribbon widths are increased with increasing molecular weight (Fig. 1H). However, the DR ratios are reduced with increasing molecular weight but exceed a value of 10 for all molecular weights, indicating the ribbons of lower molecular weight PBTTT are better aligned than high *M*_n_ PBTTT.

We observed a p-type to n-type thermopower transition in PBTTT at high doping levels. In Fig. 2A, this perpendicularly aligned $M_{1}^{⊥}$ device exhibits a positive thermopower at low |*V*_G_|, which decreases rapidly with increasing *V*_G_ and doping level, while the conductivity is enhanced with rising carrier concentration. This behavior is consistent with previous literature. However, unlike earlier studies, where the conductivity plateaus and the thermopower exhibits a small, positive, and constant value, the $M_{1}^{⊥}$ thermopower continues to decline, crossing the zero point to become negative at *V*_G_ = −2.15 V. At this transition point, the electrical conductivity reaches a maximum of 129.5 S cm^−1^ and then begins to drop. This behavior is reminiscent of the band-filling behavior recently observed in other polymer OECTs, such as indacenodithiophene (*16*); crossing the half-filling point of the highest occupied molecular orbital-derived band is associated with a peak in the conductivity and a sign change of the Seebeck coefficient. In PBTTT, it is difficult to cross the half-filling point because the lamellar polymer microstructure favors a maximum of one dopant counterion per repeat unit (*4*), as illustrated in Fig. 1D, but in contrast to our previous experiments, here we apply higher gate voltages, which force a doping level slightly above half-filling. When applying even higher gate voltages, the magnitude of the n-type thermopower peaks at −2.5 μV K^−1^ (*V*_G_ = −2.5 V) before saturating at −1.2 μV K^−1^ (*V*_G_ = −3.0 V). The conductivity drops sharply at *V*_G_ < −2.5 V; this is irreversible and suggests the onset of structural or electrochemical degradation rather than continuous band filling, which is also consistent with the thermopower saturating while the conductivity decreases.

**Fig. 2.**
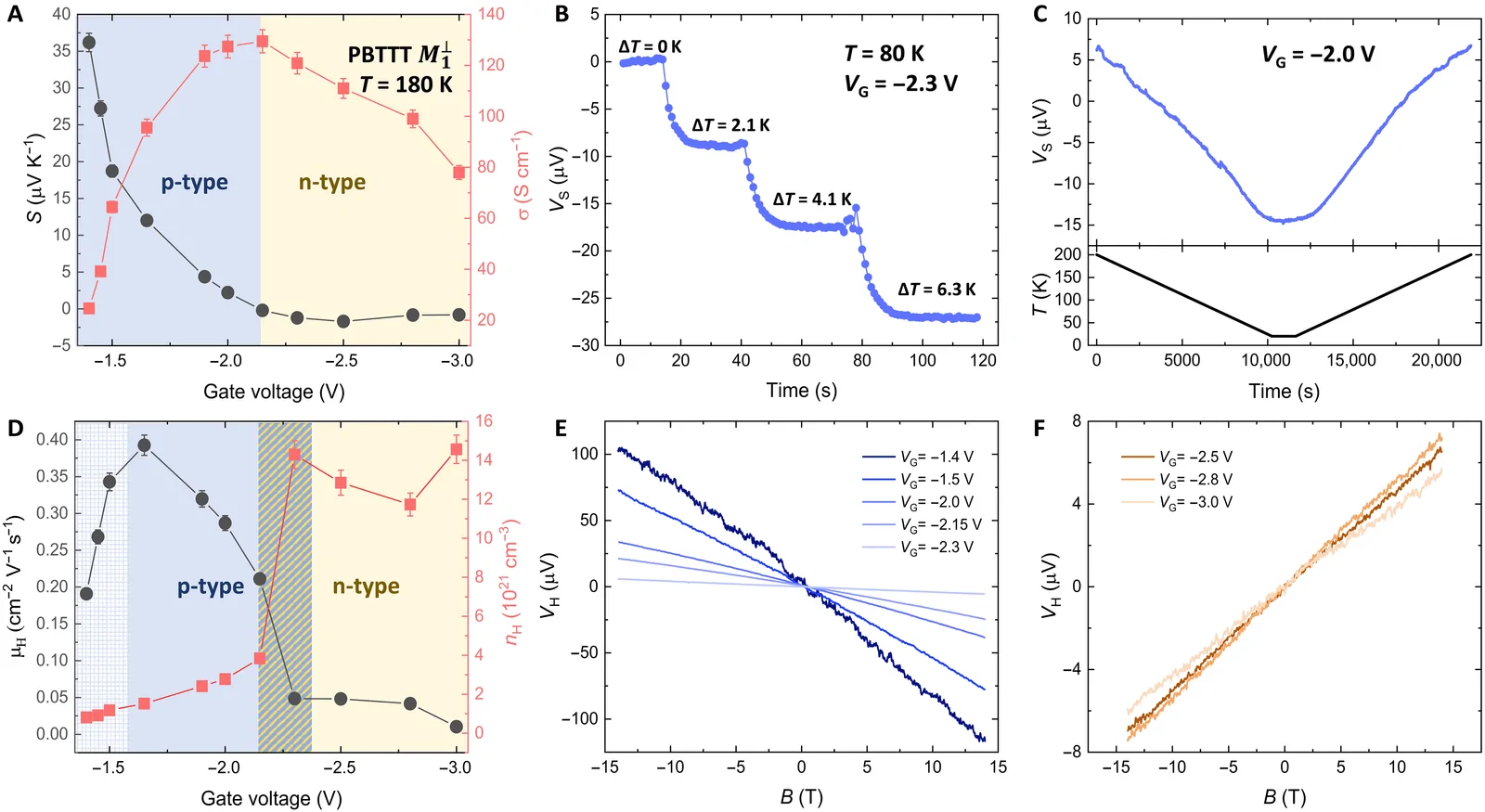
Sign changes of the thermopower and Hall effect in perpendicularly aligned PBTTT *M*_1_ OECTs. (**A**) Thermopower (*S*) and electrical conductivity (σ) as a function of gate voltage (*V*_G_) measured at 180 K. The blue shaded area denotes the p-type region (*S* > 0), while the yellow shaded area (beginning at *V*_G_ = −2.15 V) denotes the n-type region (*S* < 0). (**B**) Time trace of the n-type Seebeck voltage (*V*_S_) signal under varying temperature gradients (Δ*T*), recorded at 80 K and *V*_G_ = −2.3 V. (**C**) Time trace demonstrating the reversible p-n thermopower transition as a function of temperature (200 to 20 K) at a fixed doping level (*V*_G_ = −2.0 V). (**D**) Corresponding Hall mobility (μ_H_) and carrier concentration (*n*_H_) versus *V*_G_ at 180 K. The dashed region indicates the regime, where Hall effect and Seebeck coefficient have opposite signs, and the grid-filled region highlights the regime, where μ_H_ increases with doping (up to *V*_G_ = −1.65 V). (**E** and **F**) Linear Hall voltage plots versus applied magnetic field for p-type and n-type regimes, respectively, at selected *V*_G_ values from (D). Error bars represent one SD, derived from film thickness uncertainty (for σ) and electromotive force extraction uncertainty (for *S* and Hall measurements).

The values of the n-type Seebeck coefficient are small, but the reliability of the measurements is confirmed by the time trace of the raw data of the thermal voltage *V*_S_ (Fig. 2B; measured in situ at 80 K and *V*_G_ = − 2.3 V), which reveals a clean, linear dependence between *V*_S_ and Δ*T*, yielding a thermopower of −4.4 μV K^−1^. Potential contributions from the Au electrodes can be ruled out, as the absolute Seebeck coefficient of gold remains a small and positive value at 180 K and only becomes slightly negative ranging from −0.2 to −0.5 μV K^−1^ at 80 K (*18*, *19*). Further validation is provided by the reversible temperature dependence of the thermal voltage. When cooling a device gated at *V*_G_ = −2.0 V, i.e., just before the p-to-n transition in Fig. 2A, from 200 to 20 K and warming it up again, we observe a reversible sign transition in the thermal voltage as a function of temperature (Fig. 2C). The thermopower switches from positive at 200 K to negative at 20 K and fully recovers upon returning to 200 K, demonstrating the stability of the OECT platform. Even after electrochemical doping at high *V*_G_ values, the polymer-aligned ribbon-phase microstructures are well maintained, as revealed by AFM measurements after removing the ion gel (fig. S6).

Complementing the Seebeck data, we observed an accompanying p-type to n-type Hall effect transition in the same $M_{1}^{⊥}$ device (Fig. 2D and fig. S5). Figure 2 (E and F) shows linear Hall voltage plots versus magnetic field. At low voltages (*V*_G_ > −1.65 V), both *n*_H_ and μ_H_ calculated using the conventional expression for the Hall coefficient, *R*_H_ = (*n*_H_*q*)^−1^, increase with doping. However, as *V*_G_ increases further, *n*_H_ begins to diverge as the Hall voltage vanishes, while μ_H_ starts to decrease. At *V*_G_ = −2.3 V, the Hall coefficient drops to near zero, resulting in a nonphysical *n*_H_ value on the order of 10^22^ cm^−3^. By *V*_G_ = −2.5 V, the Hall voltage versus magnetic field reverses slope, confirming a transition to n-type Hall transport. The stability of our Hall effect measurements is demonstrated by consistent signal upon reversal of the magnetic field scan (fig. S7). These observations strongly suggest that the overestimation of carrier density from the Hall effect is closely associated with the sign reversal in the Hall and Seebeck coefficients and is likely to be a band-filling rather than a screening phenomenon; for the latter, these sign changes are not expected. We also note that the p-n switching points for Hall and Seebeck measurements differ slightly: The thermopower becomes negative at *V*_G_ = −2.15 V, while the Hall effect transition occurs between −2.3 and −2.5 V.

We observed such sign changes in Hall effect with increasing doping levels across all PBTTT OECT devices, regardless of alignment orientation (parallel versus perpendicular) or molecular weight (*M*_1_, *M*_2_, and *M*_3_). As shown in Fig. 3 (A to C), the *R*_H_ for all devices transition from positive to negative at high *V*_G_ values (figs. S5, S8, and S9). The doping-level dependence of electrical conductivity (σ) remains consistent across these variants: σ initially increases with *V*_G_, peaking near the *R*_H_ sign-reversal point, before decreasing because of a combination of crossing the half-filling point and electrochemical degradation. As expected, the peak conductivities are higher for parallel than for perpendicular devices and increase with molecular weight, particularly for the parallel devices. However, for a given molecular weight, the perpendicular and parallel devices yield nearly identical *R*_H_ values at the same *V*_G_. This suggests that they have similar doping levels and carrier densities. Even when comparing different molecular weights, the *R*_H_ values remain similar at equivalent gate voltages. For example, at *V*_G_ = −1.5 V, the *R*_H_ for $M_{1}^{⊥}$, $M_{1}^{∥}$, $M_{3}^{⊥}$, and $M_{3}^{∥}$ devices are 5.32, 5.91, 5.63, and 5.90 × 10^−9^ m^3^ C^−1^, respectively. These results indicate that the large differences in peak electrical conductivity, ranging from a maximum of 129.9 S cm^−1^ for $M_{1}^{⊥}$ to 2801.1 S cm^−1^ for $M_{3}^{∥}$, are primarily driven by differences in charge mobility. Parallel alignment and higher molecular weight substantially enhance mobility, indicating that intrachain transport along the polymer backbone is more efficient than interchain transport.

**Fig. 3.**
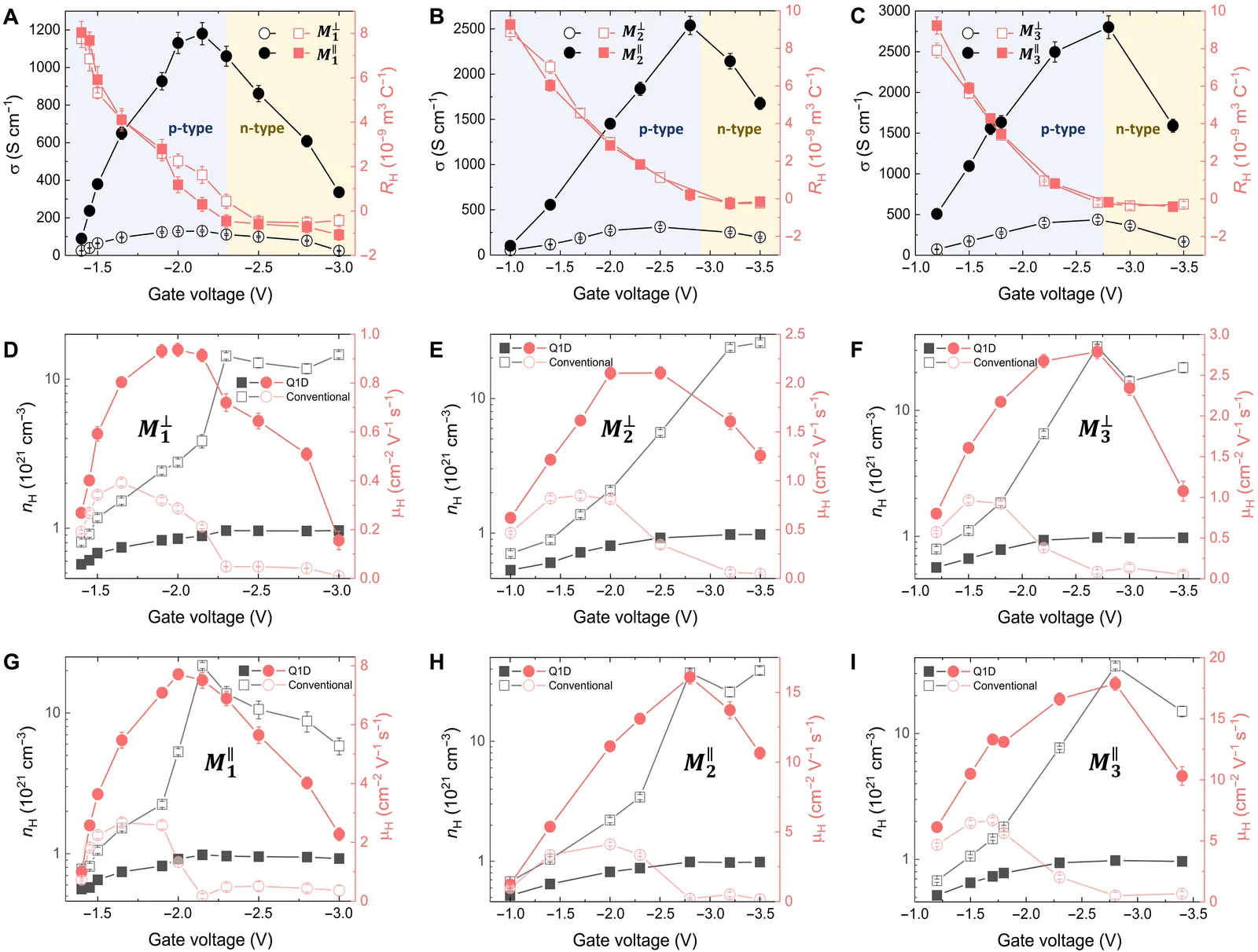
Q1D Hall effect analysis in PBTTT OECTs of different molecular weights and alignment directions. (**A** to **C**) Electrical conductivity (σ; black circles) and Hall coefficient (*R*_H_; red squares) versus gate voltage (*V*_G_) for *M*_1_, *M*_2_, and *M*_3_ devices, comparing perpendicular (open symbols) and parallel (solid symbols) orientations. Blue and yellow shaded regions denote p-type and n-type Hall regimes, respectively, showing universal sign reversal at high doping levels. (**D** to **I**) Extracted Hall carrier density (*n*_H_; black squares) and Hall mobility (μ_H_; red circles) as a function of *V*_G_, comparing the Q1D (solid symbols) and conventional (open symbols) models for all six device configurations: (D) $M_{1}^{⊥}$, (E) $M_{2}^{⊥}$, (F) $M_{3}^{⊥}$, (G) $M_{1}^{∥}$, (H) $M_{2}^{∥}$, and (I) $M_{3}^{∥}$. The conventional model yields unphysical, diverging *n*_H_ values and suppressed μ_H_ at high *V*_G_, whereas the Q1D model reveals a physically consistent saturation of carrier density near the expected limit of one charge per monomer. Error bars represent one SD determined from the uncertainty in film thickness and Hall coefficient extraction.

In the analysis of the Hall mobility and carrier density, we have so far followed the common approach, which relies on the relation for the measured Hall coefficient $R_{H}={\left(n_{H}q\right)}^{-1}$, with mobility subsequently extracted from the measured four-point probe electrical conductivity via $\sigma =n_{H}q\mu_{H}$. As shown above, this method yields unphysically high carrier densities and therefore underestimates charge mobilities, near the point where Hall effect and Seebeck coefficient change sign. In principle, this should not be unexpected because the $R_{H}={\left(n_{H}q\right)}^{-1}$ is only valid at relatively low doping levels, whereas our uniaxially aligned polymers are highly doped and exhibit a highly anisotropic band structure with strong intrachain dispersion governed by a large intrachain transfer integral *t*_*x*_ and weaker interchain dispersion governed by the smaller interchain transfer integral *t*_*y*_ (*20*). The band structure in the two-dimensional transport plane parallel to the substrate might more accurately be represented by a Q1D tight binding band with dispersion

$$\epsilon \left(k_{x},k_{y}\right)=-2t_{x}\text{cos}\left(k_{x}a\right)-2t_{y}\text{cos}\left(k_{y}b\right)$$ (1)

where *a* and *b* are the monomer length along the backbone and the π-π stacking distance, respectively, and *k*_*x*_ and *k*_*y*_ are the corresponding wave vectors. By applying the Boltzmann transport formalism to this Q1D tight-binding band structure (*21*, *22*) and calculating the Hall conductivity σ_*xy*_ from an integral of the group velocities over the Fermi surface across the Brillouin zone, the Hall coefficient can be expressed as a function of band-filling factor *f* (see Supplementary Text)

$$R_{H}=\frac{1}{n_{H}q}f$$ (2)

$$f=\frac{k_{F}a}{\text{tan}\left(k_{F}a\right)}$$ (3)

Here, *k*_F_ is the Fermi wave vector, which is related to the three-dimensional charge density by

$$k_{F}=\frac{\pi \mathrm{bc}}{2}n_{H}$$ (4)

Here, *c* is the lamellar stacking distance. At low filling (kFa≪π2), the expression of *R*_H_ reduces to the conventional formula $R_{H}=\frac{1}{n_{H}q}$, and *R*_H_ is positive. At the half-filling state (kFa=π2), *R*_H_ is predicted to be zero, and beyond half-filling (kFa>π2), *R*_H_ will become negative. These predictions of the model are in good qualitative agreement with our observations.

To provide a more quantitative analysis, we applied the Q1D model to extract charge carrier mobility and density from the PBTTT Hall effect data (Fig. 3, A to C) and compared the results with the conventional analysis ($R_{H}={\left[n_{H}q\right]}^{-1}$). On the basis of previous structural studies, the unit cell dimensions for PBTTT were taken as *a* = 13.5 Å, *b* = 3.7 Å, and *c* = 20.2 Å (*4*, *20*, *23*), which correspond to a theoretical carrier density of 9.91 × 10^20^ cm^−3^ for a doping level of one charge per monomer. The extracted values for all PBTTT devices (*M*_1_, *M*_2_, and *M*_3_ in perpendicular and parallel configurations) are plotted in Fig. 3 (D to I). For both the conventional and Q1D model, the extracted *n*_H_ increases with doping level and *V*_G_. However, for the conventional model, the *n*_H_ values are generally larger than those from the Q1D model. Furthermore, the rate of increase for conventional *n*_H_ accelerates continuously as *R*_H_ approaches zero, eventually diverging to unphysical values in the 10^22^ cm^−3^ regime, implying more than 10 charges per monomer. This also leads to the erroneous conclusion that the doping level cannot be saturated, although the electrical conductivity (Fig. 3, A to C) has already reached its maximum. In contrast, the Q1D model reveals carrier densities that saturate at high gate voltages, with consistent maximum *n*_H_ values of 9.64, 9.82, 9.76, 9.86, 9.79, and 9.80 × 10^20^ cm^−3^ for $M_{1}^{⊥}$, $M_{2}^{⊥}$, $M_{3}^{⊥}$, $M_{1}^{∥}$, $M_{2}^{∥}$, and $M_{3}^{∥}$ devices, respectively. These values are in excellent agreement with the expected charge concentration calculated from the one charge per monomer limit and also agree with previous XPS findings (*4*), as illustrated in Fig. 1D. The fact that the Hall carrier densities agree so consistently with what is known to be the “true” carrier density of PBTTT in this high doping regime provides strong evidence of the validity of our proposed model.

Regarding the extracted μ_H_, the Q1D model also provides more reasonable values than the conventional analysis: In both models, μ_H_ initially increases with doping but eventually decreases because of overdoping. However, the peak in μ_H_ appears at very different doping levels. In the conventional model, μ_H_ reaches its maximum before the peak in conductivity. Numerically, this is because the diverging *n*_H_ (>10^22^ cm^−3^) at high gate voltages suppresses μ_H_, even when conductivity is high. For example, at *V*_G_ = −2.8 V, the $M_{2}^{∥}$ device exhibits a maximum conductivity of 2538 S cm^−1^ but a conventional μ_H_ of only 0.4 cm^−2^ V^−1^ s^−1^ (Fig. 3H). This low mobility is an underestimation for aligned PBTTT, which is a metallic system with relatively high crystallinity. Conversely, the Q1D model provides a more physical result: The maximum in μ_H_ becomes much closer to the maximum in conductivity. For the $M_{3}^{∥}$ device, the Q1D model yields a peak mobility of 17.9 cm^−2^ V^−1^ s^−1^ at V_G_ = −2.8 V, approximately 35 times larger than the conventional value.

To further validate our Q1D model, we performed OECT gate charging current analysis, which involved integrating the gate current (*I*_G_) over time to estimate the total number of injected charges at a given gate voltage. By assuming that each injected ion creates a free carrier, this provides an independent measurement of the carrier density that can be compared to that derived from the Hall coefficient (figs. S10 and S11). To ensure accuracy, we precisely patterned both the polymer channel and the ion gel to minimize the contact area of the ion gel with the electrodes. As shown in Fig. 4A, the carrier density determined from gate current integration is consistent with the Q1D model, also yielding a maximum density of ∼1 × 10^21^ cm^−3^ (one charge per monomer), while the conventional model again yields diverging carrier density values exceeding 3 × 10^22^ cm^−3^. These results provide a compelling validation of our Q1D model for interpreting the Hall data and suggest that it provides a reliable and accurate estimation of the true carrier density across a wide range of doping levels, even beyond half-filling.

**Fig. 4.**
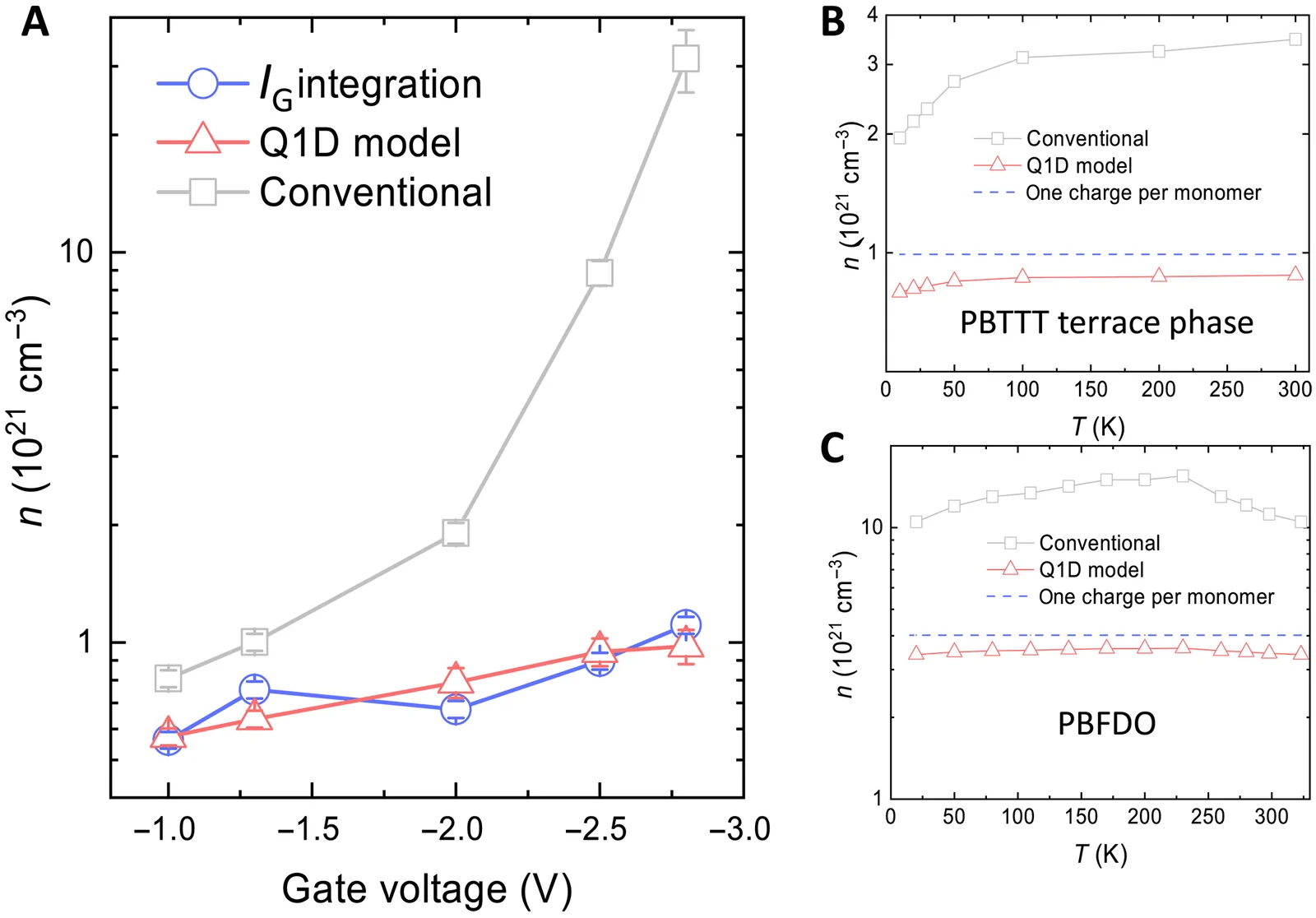
Experimental validation of the Q1D model and band-filling picture of the observed bipolar Hall and Seebeck effect. (**A**) Comparison of carrier density (*n*) versus gate voltage (*V*_G_) determined via three independent methods for an aligned $M_{3}^{∥}$ PBTTT OECT: in situ experimental estimation from gate-current integration over time (*I*_G_ integration; blue open circles), extraction from Hall coefficients using the Q1D metallic model (red open triangles), and extraction using the conventional model (gray squares). The Q1D model achieves excellent agreement with the experimental *I*_G_ integration data. Error bars represent one SD determined from the uncertainty in film thickness and Hall coefficient extraction. (**B** and **C**) Reanalysis of published Hall effect data in spin-coated terrace phase PBTTT and PBFDO with the Q1D model (*3*, *24*), respectively.

We then attempted to apply our Q1D model of *R*_H_ to Hall measurements reported in literature and found that it also provides a good approximation for interpreting the Hall effect, even in systems with less structural anisotropy (Supplementary Text). We chose spin-coated films of PBTTT and PBFDO as model systems and reanalyzed published Hall data using our Q1D model (*3*, *24*). As shown in Fig. 4 (B and C), the Q1D model gives more physically reasonable carrier densities for heavily doped films, approximately one charge per monomer, confirming the generality of its substantially improved accuracy. The extracted carrier density is approximately temperature independent, which is also more reasonable than the temperature-dependent carrier density implied by the conventional analysis. In Supplementary Text, we discuss why our Q1D model provides more reliable estimates of carrier densities also for systems, in which the polymer chains are not uniaxially aligned but randomly oriented in the transport plane. The reason is that in the regime near half-filling, the predicted values for *R*_H_ do not vary strongly with the anisotropy in the transfer integrals (fig. S14): For an arbitrary ratio $\frac{t_{y}}{t_{x}}$ no analytical expression could be derived for *R*_H_, but the extracted values for carrier density remain within 20% of those predicted by the Q1D model that assumes *t*_*x*_ ≫ *t*_*y*_. The PBTTT in Fig. 4B is chemically doped (*3*), and the PBFDO in Fig. 4C is self-doped (*24*). The successful quantitative interpretation of their Hall data demonstrates that our Q1D transport model is broadly applicable to polymers doped via different methods.

We evaluated the *S* ∼ log(σ) relationship for all PBTTT OECT devices (fig. S12) and observed a consistent slope independent of molecular weight and alignment direction before reaching the maximum electrical conductivity. We also calculated the thermoelectric power factor *PF* = *S*^2^σ, which was found to be increasing with molecular weight and higher for parallel than for perpendicular orientation; it reached a maximum value of 162.3 μW m^−1^ K^−2^ in $M_{3}^{∥}$ (fig. S13).

Last, we comment on the difference in gate voltage, at which the Hall and Seebeck coefficients change sign. On the basis of the Boltzmann transport equation, the Seebeck coefficient is not only determined by the energy-dependent density of states (DOS), *D*(*E*), but also by the energy dependence of the relaxation time τ(*E*) around the Fermi level (*E*_F_)

$$S\propto -{\left.\left(\frac{\partial \text{ln}D\left(E\right)}{\partial E}+\frac{\mathrm{\partial ln\tau}\left(E\right)}{\partial E}\right)\right|}_{E=E_{F}}$$ (5)

The first term is expected to undergo a sign reversal at half-filling similar to the Hall coefficients, while the second term depends on the actual charge scattering mechanism. Therefore, the sign switching of the Seebeck coefficient may occur at a different doping level than that of the Hall coefficient (Fig. 2). In Supplementary Text, we also comment on why the Seebeck coefficient does not exhibit different signs for the parallel and perpendicular transport directions, which would be expected in the Q1D model from simple DOS and group velocity considerations. To explain this, more sophisticated transport models are needed that take into account the contribution from τ(*E*).

## DISCUSSION

In this study, we used sign change observations in both the Hall coefficient and thermopower in aligned PBTTT OECTs upon p-doping to demonstrate that the widely reported overestimation of Hall carrier density in conducting polymers is not a result of the screening effect by localized charge carriers. Rather, it stems primarily from the band-filling effect and the fact that conventional interpretation of the Hall effect (*R*_H_ = [*n*_H_*q*]^− 1^) is inapplicable for heavily doped polymers. We propose a tight-binding Q1D metallic model that provides an analytical expression to explain the observed sign change in Hall effect data and to provide reliable estimates of charge carrier density. The values obtained are consistent with other measurements and correspond to a saturation doping level of one charge per monomer incorporated into the lamellar microstructure of PBTTT. Our findings show that a simple band structure framework considering the DOS and group velocity at the Fermi energy across the Brillouin zone provides an adequate interpretation of the Hall response of these materials. This is somewhat unexpected given the complex, correlated electron physics of these highly doped systems, which is subject also to strong electron-electron, electron-ion, and electron-phonon coupling effects (*25*). Why this is possible may need to be better understood, and the applicable limit of the Q1D model requires further study. Poly(3-hexylthiophene) exhibits large interchain delocalization (*26*) and observable Hall effect, but thermally activated conductivity, which could be an ideal candidate to test the limit of the Q1D framework. From a practical perspective, our work provides a reliable and easily applicable methodology for extracting carrier density and mobilities from the Hall effect in high-performance conducting polymers, which is generally applicable to a wide range of near-metallic polymer systems.

## MATERIALS AND METHODS

### Materials

PBTTT-C12 (poly(2,5-bis(3-dodecylthiophen-2-yl)thieno[3,2-b]thiophene)) (1-Material), (see the Supplementary Materials for detailed information), 1,2-dichlorobenzene [anhydrous DCB; ROMIL Hi-Dry; <20–parts per million (ppm) water], BMP TFSI (Solvionic; 99.9%, <20–ppm water and <1–ppm halides), PVDF-HFP (Sigma-Aldrich), and acetone (Thermo Fisher Scientific).

### PBTTT films

PBTTT solutions (10 mg ml^−1^, DCB) were heated at 120°C for 1 hour before use. The substrates for PBTTT film deposition are based on Corning glass (EAGLE XG) with patterned Hall bar, heater, thermometer, and side gate electrodes, which were fabricated by standard process of photolithography, Cr/Au deposition (5/30 nm), and lift-off. After the cleaning of substrates by ultrasonication and O_2_ plasma, the aligned PBTTT films were prepared using a home-built blade coater with a polytetrafluoroethylene (PTFE) rod as the coating blade. A total of 10 μl of the polymer solution was injected into the gap between the blade and the substrate. Then, the temperature-controlled substrate (80°C) moved at a fixed speed of 110 μm s^−1^ underneath a PTFE coating blade, in directions either parallel or perpendicular to the Hall bar channel for different chain alignment orientations. After blade coating, the deposited polymer on the substrate was annealed at 270°C for 20 min and then cooled down to room temperature on hotplates naturally. All solution preparation, film deposition, and annealing processes were performed in a glovebox under nitrogen atmosphere (MBRAUN; <1-ppm O_2_ and <1-ppm H_2_O).

### OECT Hall bar device fabrication

After the deposition of aligned PBTTT films, the samples were patterned into well-defined Hall bar geometry by a laser ablation system. An ion-gel layer was then spin coated on top of the patterned polymer films (5000 rpm for 30 s). Only the ion gel that covers the side gate and the polymer channel is required to be remained, while the ion gel on the contact pads was peeled off to ensure good electrical connection in following measurements. The ion-gel solution was prepared by mixing PVDF-HFP, ionic liquid BMP TFSI, and acetone with a weight ratio of 1:4:20. Last, the OECT Hall bar devices were fixed onto standard PPMS pucks by GE varnish and connected by spot welding using aluminum wires.

### Transport measurements

The electrical conductivity, Seebeck, and Hall effect measurements on the patterned and electrochemical gated devices were carried out in situ in vacuum (10^−2^ torr) with a Quantum Design Physical Property Measurement System (PPMS, DynaCool-14T). The input electrical signals to the OECT Hall bar devices are applied by a Keithley 2612B source measurement unit, while the voltage signals are measured by a Keithley 2182A nanovoltmeter with an input impedance of 10 gigohms. The wirings outside the PPMS chamber were custom made low-noise coaxial cables, which results in an overall electrical noise of less than 10 nV when the system is shorted. Most transport measurements in this study were performed at 180 K to freeze the ion gel and the electrochemical doping state for stable transport measurements. Specifically, the electrochemical doping was performed at 300 K by gating at certain gate voltages for 30 min to reach equilibrium, and then the PPMS environment temperature was decreased from 300 to 180 K at a speed of 3 K/min while gating. No drain voltage was applied during cooling. Once the temperature reached 180 K, the ion gel was frozen, and the gate voltage was then removed for subsequent transport measurements. Below 180 K, the change of temperature requires to be more careful with a slower speed of 1 K/min. After the measurements at 180 K, the temperature was increased back to 300 K to change the electrochemical doping state by applying different gate voltages. The PPMS built-in ±14T superconducting magnetic field in the perpendicular direction with a sweeping speed of 100 Oe/s is used for Hall effect measurements, in which a current was applied across the channel from source to drain while the Hall voltages were measured by the transverse probes. For Seebeck measurements, the temperature gradient is created by applying electrical input signals to the on-chip heater, and the calibration of the temperature difference is performed by the two thermometers close to the device channel. Detailed temperature calibration procedure and data are presented in the Supplementary Materials. The electrical conductivity measurements were performed in a standard four-point probe method.

### UV-vis-NIR absorbance

The UV-vis-NIR measurements were performed using an Agilent Cary 6000i UV-Vis-NIR spectrophotometer fitted with a tungsten halogen vis-NIR lamp and a deuterium arc UV lamp. The UV-vis-NIR spectra shown are single scans collected with an integration time of 0.025 s per point.

### AFM measurements

Tapping-mode AFM measurements were performed on an Oxford Instruments Cypher ES Environmental AFM with an RTESPA-150 probe (resonant frequency: 150 kHz, spring constant: 0.4 N m^−1^; Bruker) to characterize the surface morphology of the samples at a scan rate of 0.5 Hz and 512 samples per line. The data were analyzed with Gwyddion 2.63, including the analysis of PBTTT ribbons by fast Fourier transform.

## Supplementary Materials

### This PDF file includes:

Supplementary Text Figs. S1 to S15 References
