# Supplementary material for: Interpreting the Hall effect of highly doped conducting polymers
Source: Sci Adv. 2026 Oct 21;12(43):eaej6501. doi: 10.1126/sciadv.aej6501 (PMC13686005; doi:10.1126/sciadv.aej6501)
Supplement: Supplementary file 1 [file sciadv.aej6501_sm.pdf]

Science Advances
AAAS

Supplementary Materials for

Interpreting the Hall effect of highly doped conducting polymers

Yingqiao Ma *et al.*

Corresponding author: Xinglong Ren, xr216@cam.ac.uk; Henning Sirringhaus, hs220@cam.ac.uk

Sci. Adv. **12**, eaej6501 (2026)
DOI: 10.1126/sciadv.aej6501

This PDF file includes:

Supplementary Text
Figs. S1 to S15
References

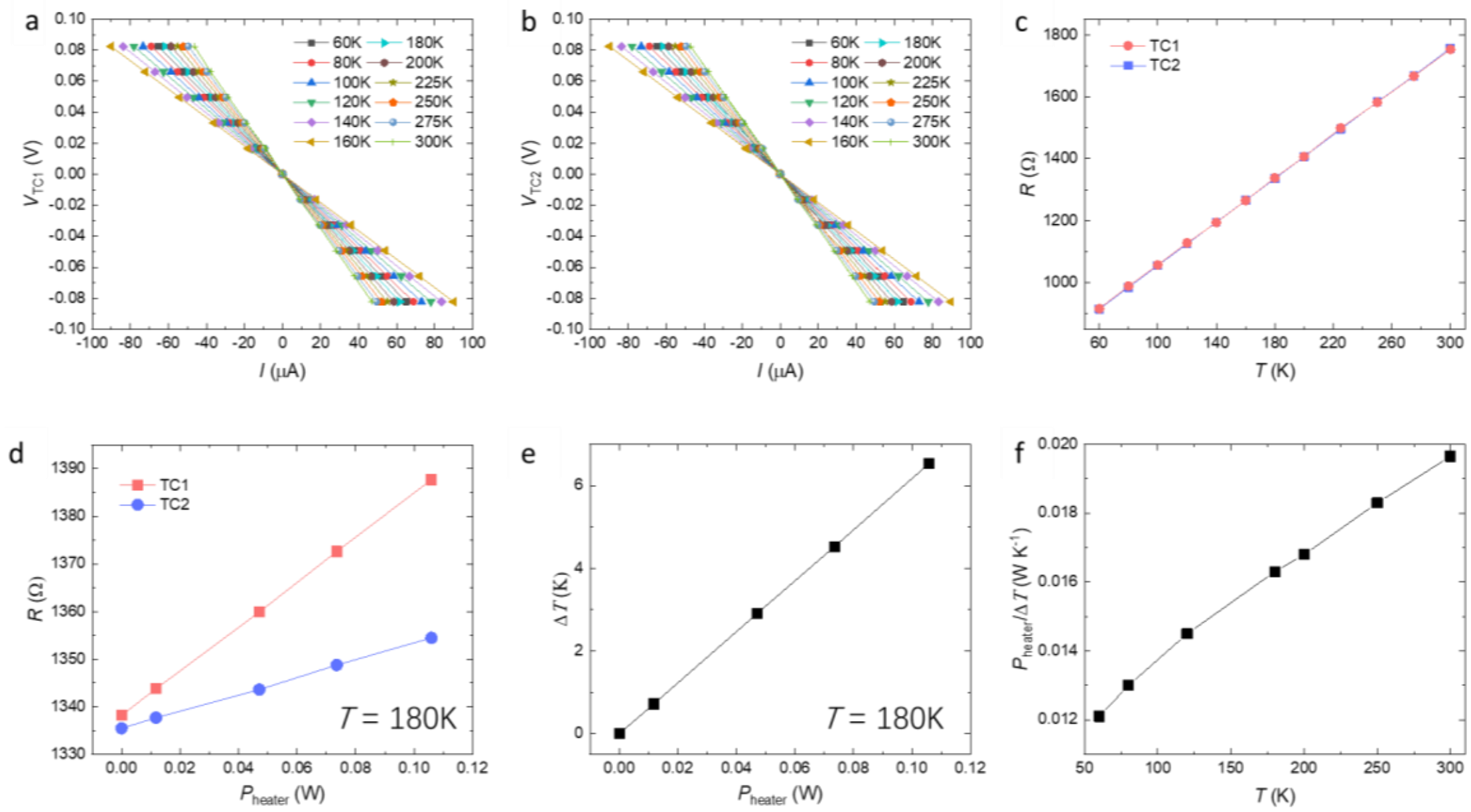

Fig. S1.

Temperature calibration of the OECT Hall bar Seebeck chip. The temperature calibration process is performed in-situ in the PPMS chamber. **(a)** and **(b)** Four-probe method measured resistance of the on-chip thermocouples at the hot end (TC1) and the cold end (TC2) of the channel versus controlled ambient temperature. **(c)** The revealed linear temperature dependence of the TC1 and TC2 resistance between 300 K and 60 K. **(d)** At the measurement temperature 180 K, the resistance of TC1 and TC2 were measured at and plotted with different powers applied to the on-chip heater. **(e)** Calibrated temperature difference (ΔT) across the patterned polymer film channel versus the heating power by combining (c) and (d) and considered the geometry of the device (the ratio between the distance from TC1 to TC2 and the channel length). **(f)** Temperature calibration at different measurement temperature. By repeating the same procedure at 180 K, the heater power to ΔT ratio is plotted versus the PPMS environment temperature.

a

	Mn (kda)	Mw (kda)	PDI
M_1	22.5	38	1.68
M_2	31.7	60.7	1.91
M_3	47.1	80	1.65

b

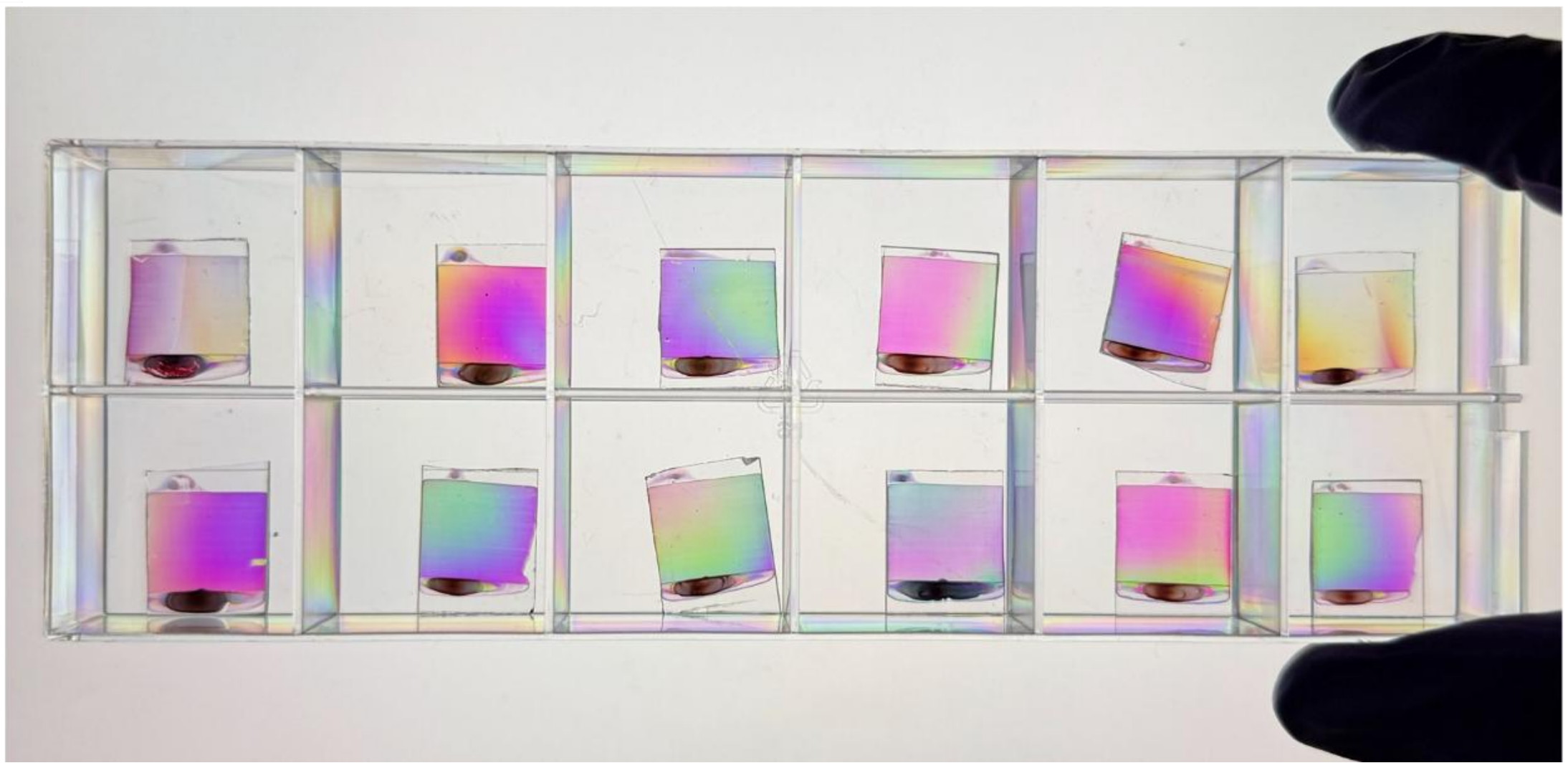

Fig. S2.

(a) Molecular weight and polydispersity index distribution data for PBTTT applied in this study. **(b)** Photo of the double refraction rainbow in ribbon phase PBTTT films fabricated via refined blade-coating procedures (*17*), indicating the PBTTT ribbons are well aligned.

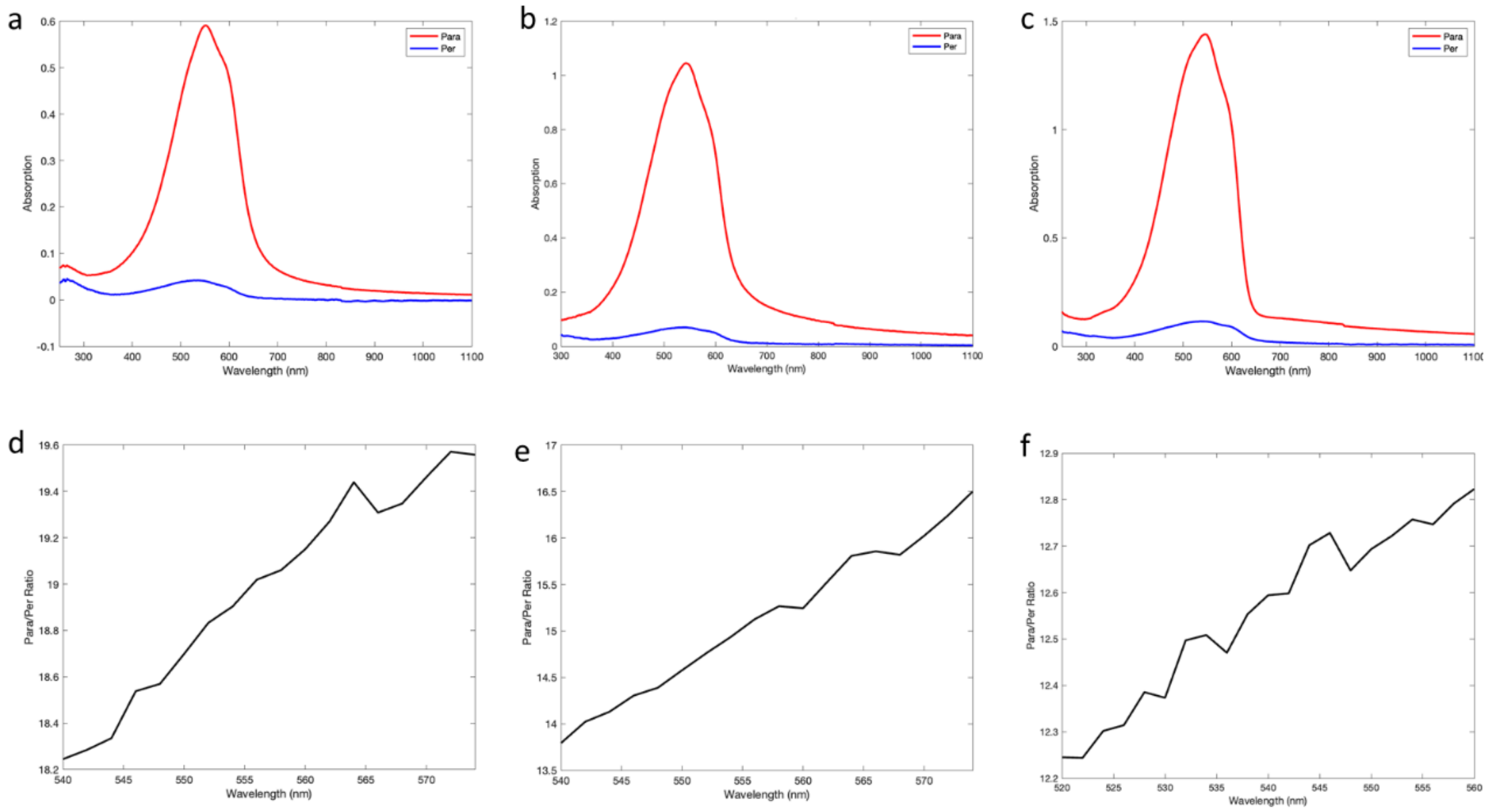

Fig. S3.

(a), **(b)** and **(c)** are the UV-Vis-NIR absorption spectra of parallel and perpendicular aligned PBTTT M_1, M_2 and M_3 films in pristine state, respectively. **(d)**, **(e)** and **(f)** are their corresponding dichroic ratio (DR) between parallel aligned and perpendicular aligned films, all revealing high DR ratios over 10, indicating good chain alignment for all cases.

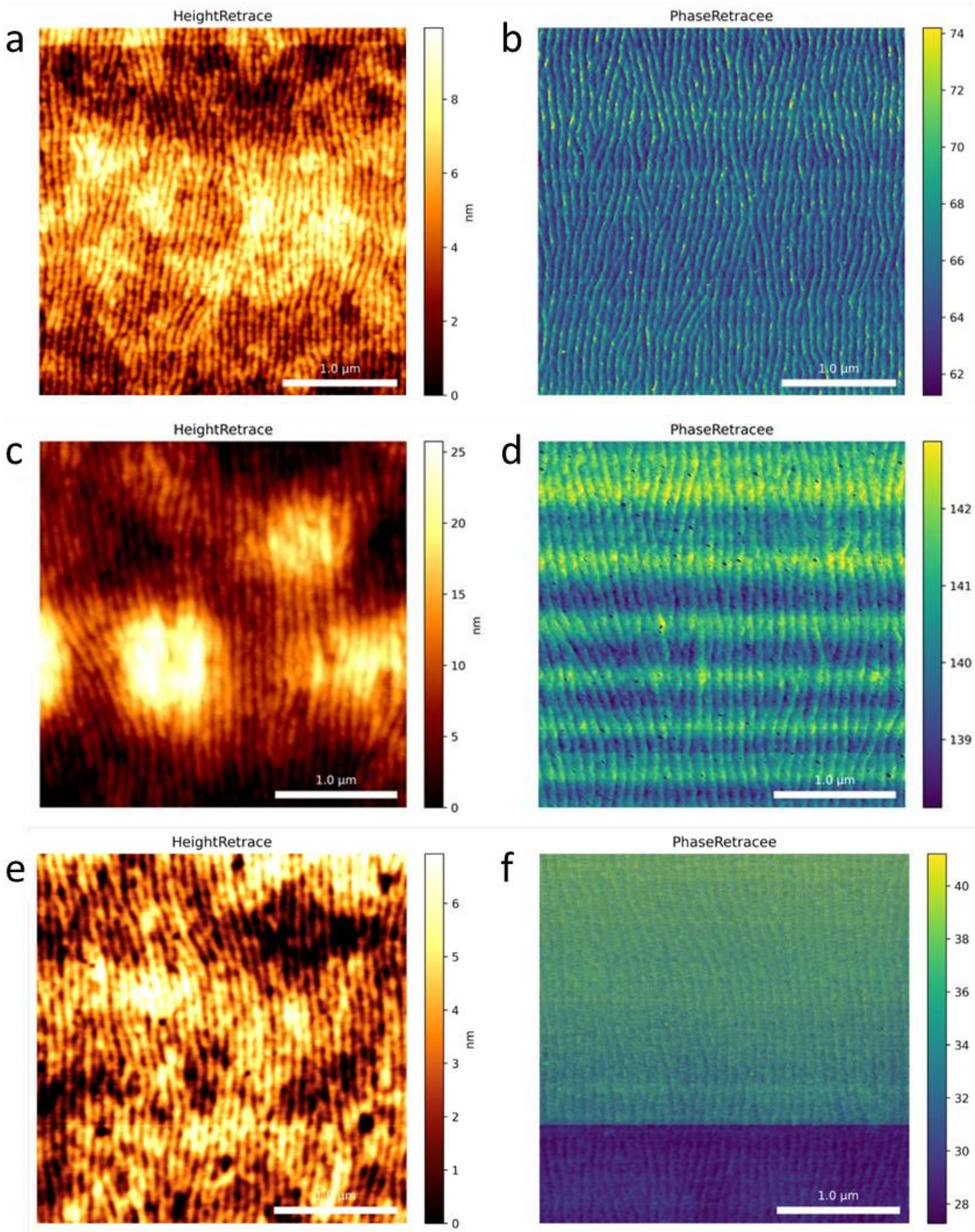

Fig. S4.

AFM topography and phase images for ribbon phase aligned PBTTT M_1, M_2 and M_3 films. **(a)** and **(b)** M_1; **(c)** and **(d)** M_2; **(e)** and **(f)** M_3, respectively. The AFM images reveal clean and highly aligned ribbon phase PBTTT for all cases, indicating high crystallinity.

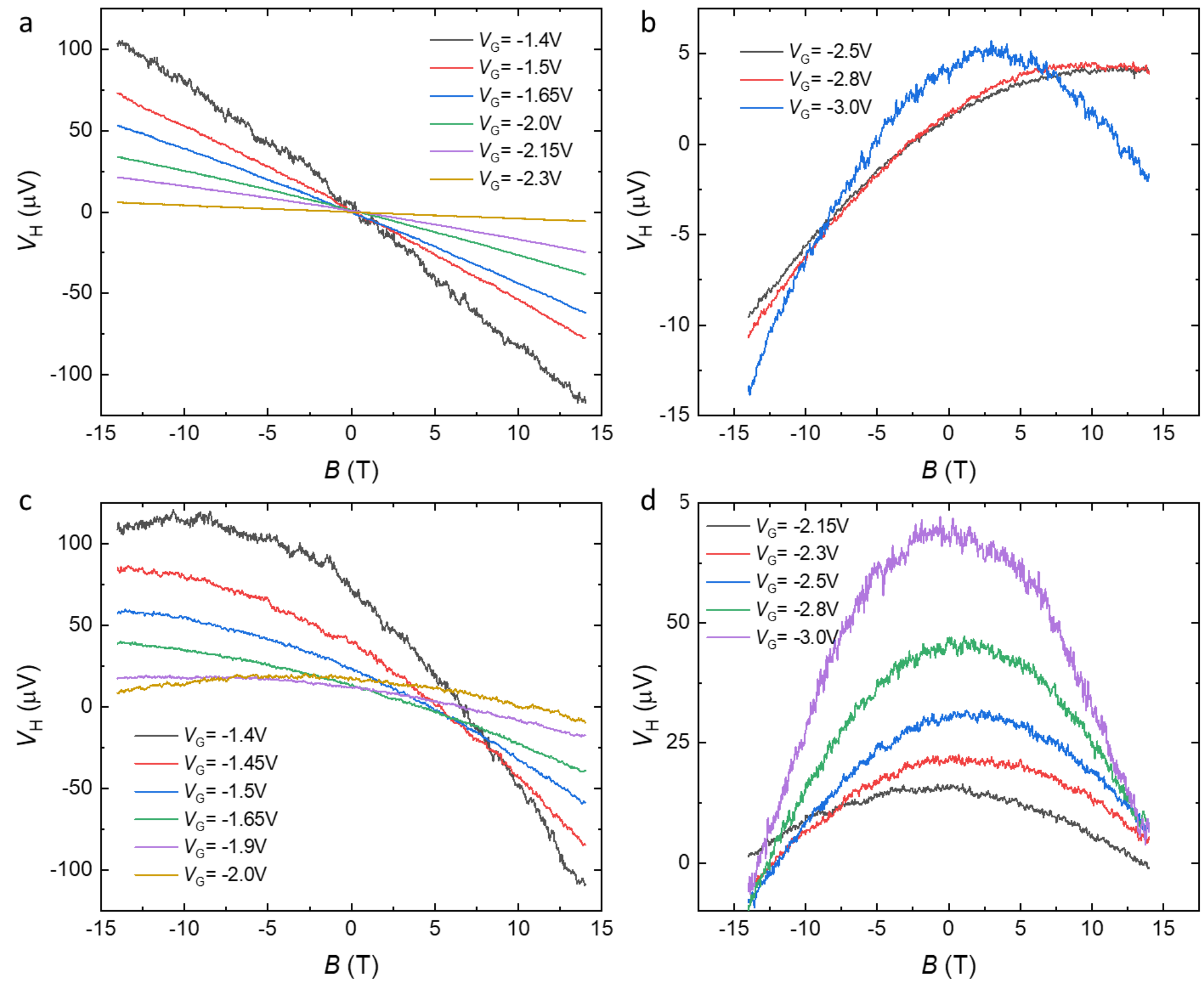

Fig. S5.

Gating voltage dependent bipolar Hall effect data of perpendicular aligned (a and b), and parallel aligned (c and d) PBTTT M_1 devices measured at 180 K. This corresponds to Fig. 3a in the main text. **(a)** For perpendicular aligned PBTTT M_1 OECT devices, with the increase of gating voltages from -1.4 V to -2.3 V, the p-type (hole dominating) Hall voltage signals gradually decreases to nearly zero with the sweeping of magnetic field and a constant source-drain current. **(b)** Once the gate voltage is further increased to -2.5 V, -2.8 V and -3.0 V, the Hall voltage signals changed the sign and became n-type (electron dominating). It should be noted that the non-linearity in the Hall effect data shown here is due to the magnetoresistance component from the miss alignment of the two transverse Hall probes, which has also been revealed in previous Hall and Nernst effect studies (*5*). The non-linear components have been extracted in a similar way as in previous studies. After extraction the data corresponds to Fig. 2f in the main text. **(c)** and **(d)** show p-type and n-type Hall effect data in the parallel aligned PBTTT M_1 device, respectively.

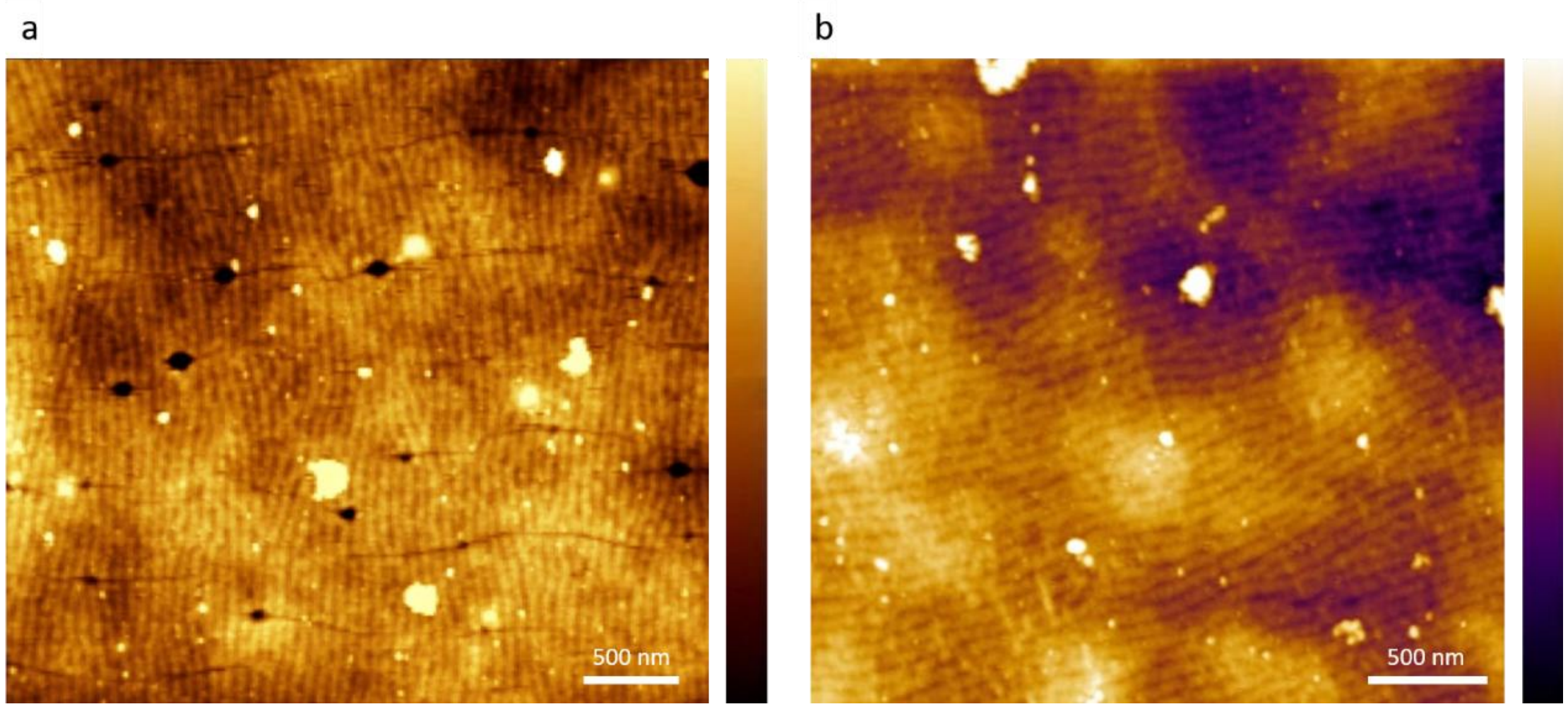

Fig. S6.

AFM images of ribbon phase PBTTT OECT devices after electrochemical gating. The BMP TFSI/PVDF-HFP ion-gel was removed by the acetone washing before AFM measurements. The bright clusters in the images are residue ion-gel. **(a)** $M_1^{\parallel}$; **(b)** $M_1^{\perp}$. It can be seen that the PBTTT ribbons remained to be well aligned after OECT measurements. The observable structure degradation features are much less as compared to previous chemical doping (*17*).

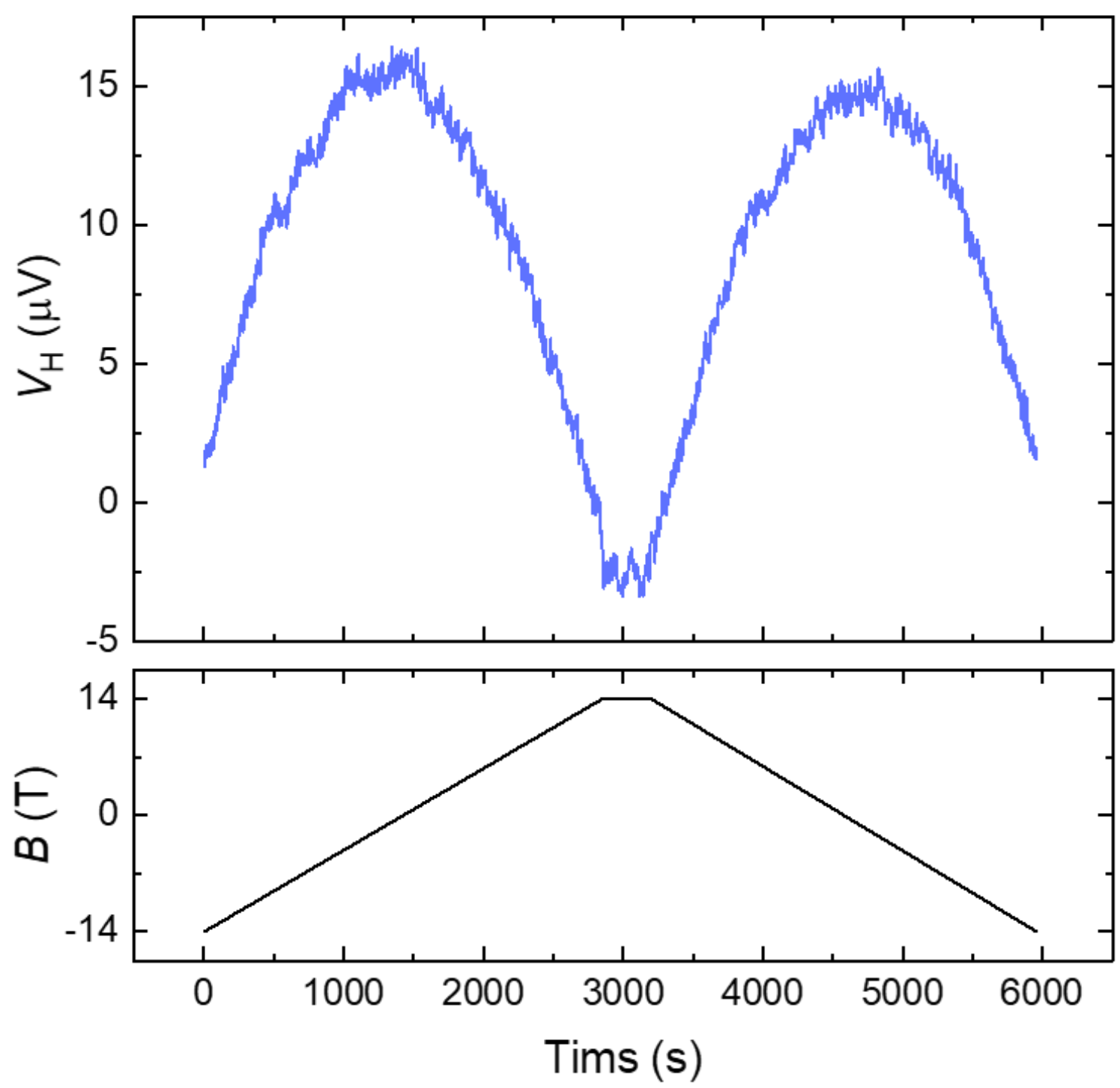

Fig. S7.

Hall effect stability test around the p-n switching point at 180 K. This is measured at a gate voltage of -2.15 V in parallel aligned PBTTT M_1 device, corresponding to Fig. S5d. At this doping state, the Hall voltage is nearly unchanged with a very small p-type signal when the magnetic field was changed from -14 T to 14 T (0-3000 s), indicating the onset of p-n switching. The magnetic field was then swept back from 14 T to -14 T (3000-6000 s), and the Hall signal was well reproduced with negligible signal drift, proving the stability of our devices. This test demonstrates that the measured n-type Hall signal at high doping level in our aligned PBTTT devices are reliable and not induced by stability issue related electrical signal drift over time, even though the magnitude of the signal is relatively small as compared to p-type Hall signal at low doping levels.

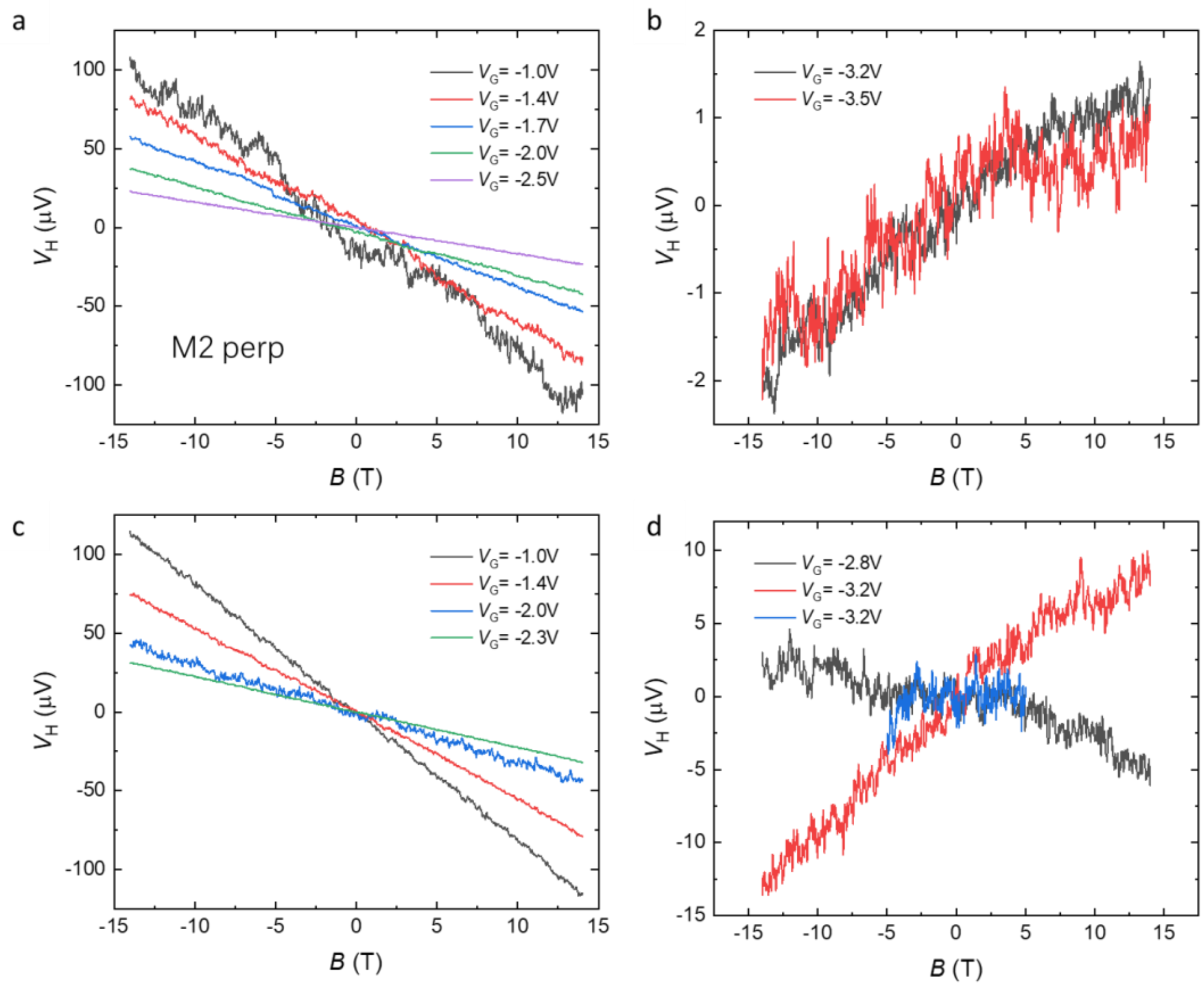

Fig. S8.

Gating voltage dependent bipolar Hall effect data of perpendicular aligned (a and b), and parallel aligned (c and d) PBTTT M_2 devices measured at 180 K. This corresponds to Fig. 3b in the main text. **(a)** and **(b)** show p-type and n-type Hall effect data in the perpendicular aligned PBTTT M_2 device, respectively. **(c)** and **(d)** show p-type and n-type Hall effect data in the parallel aligned PBTTT M_2 device, respectively.

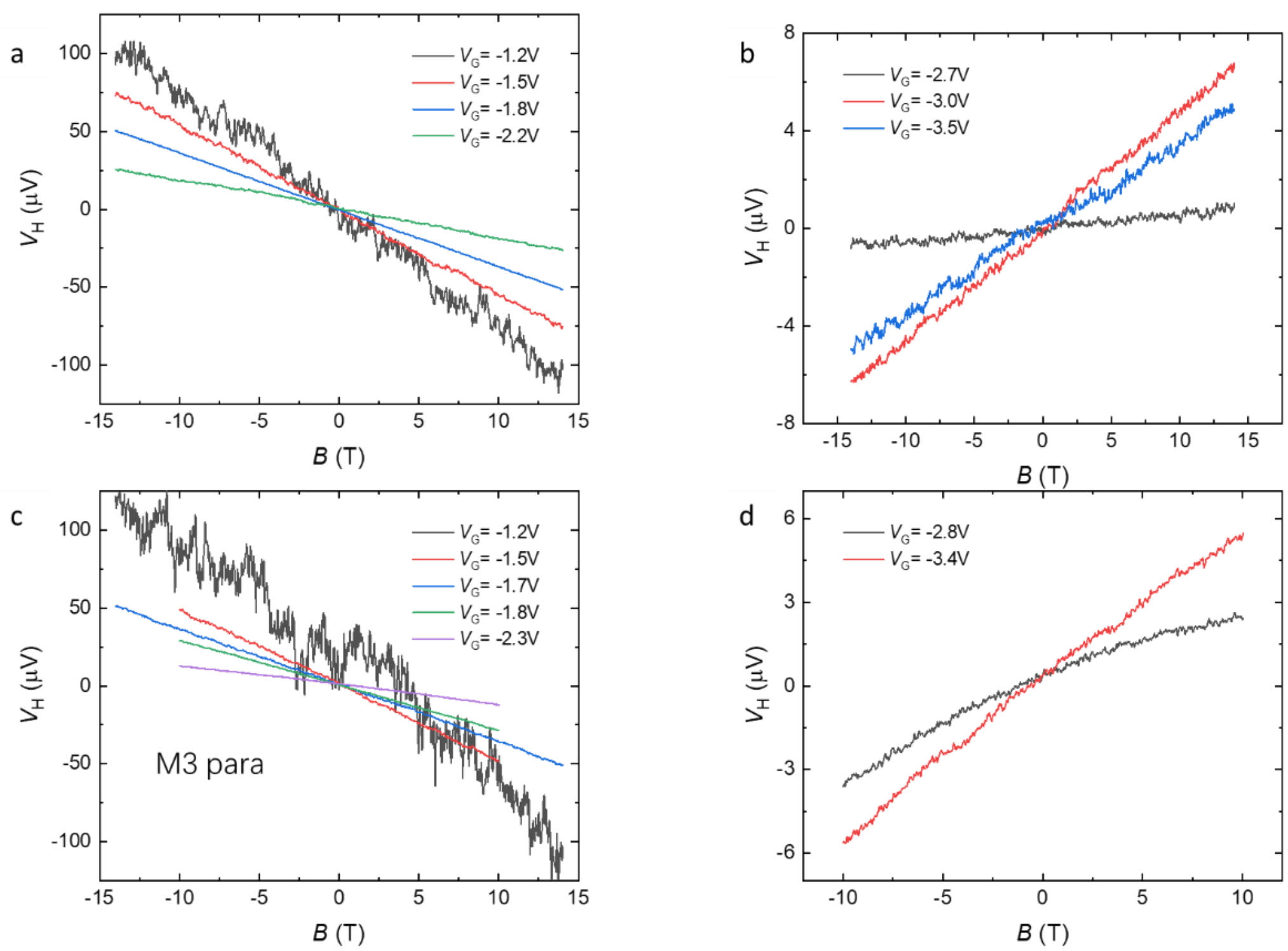

Fig. S9.

Gating voltage dependent bipolar Hall effect data of perpendicular aligned (a and b), and parallel aligned (c and d) PBTTT M_3 devices measured at 180 K. This corresponds to Fig. 3b in the main text. **(a)** and **(b)** show p-type and n-type Hall effect data in the perpendicular aligned PBTTT M_3 device, respectively. **(c)** and **(d)** show p-type and n-type Hall effect data in the parallel aligned PBTTT M_3 device, respectively.

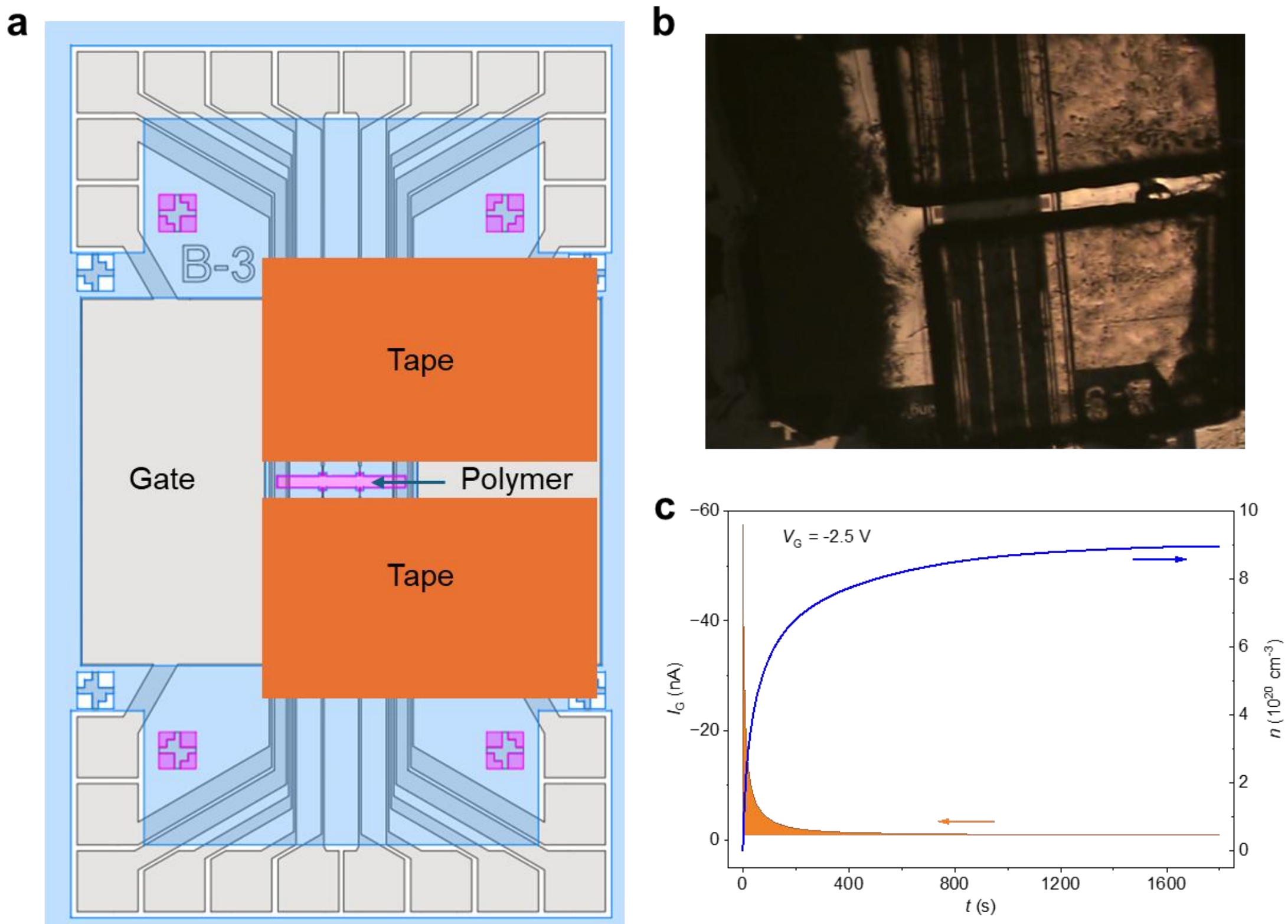

Fig. S10.

Charge carrier density estimated from gate current integration. (a) Schematic illustration of the device structure: the polymer (pink rectangle in the middle) is patterned by photolithography; most of the source/drain electrodes are covered by tape (orange rectangles) to minimize parasitic current; the ion gel is applied to cover the polymer channel and the side gate (large grey rectangle). **(b)** optical image of a device after the application of ion gel. The volume of the polymer channel is roughly 1.05*0.1*0.0001 mm^3. **(c)** Gate current and estimated carrier density as a function of charging time for V_G = -2.5 V. The orange area represents the integrated area.

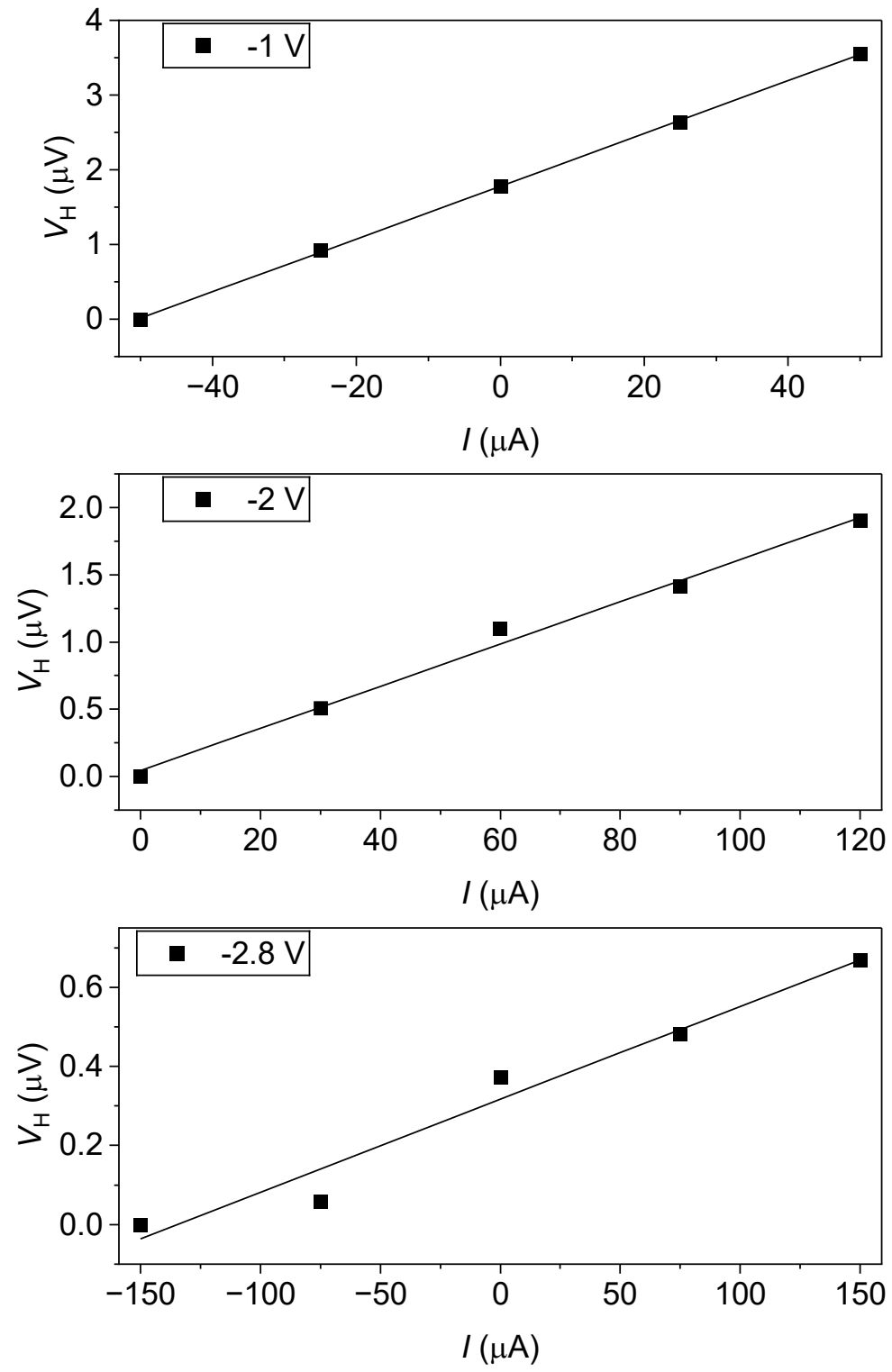

Fig. S11.

AC Hall signal. The device used for the gate current integration measurements was measured in a homemade AC Hall measurement setup. Therefore, the corresponding Hall coefficients were also acquired from AC Hall measurements. The magnetic field used here is 0.47 T and the frequency of the AC magnetic field is 1.2 Hz. Details of AC Hall measurements can be found in our previous publication (*3*).

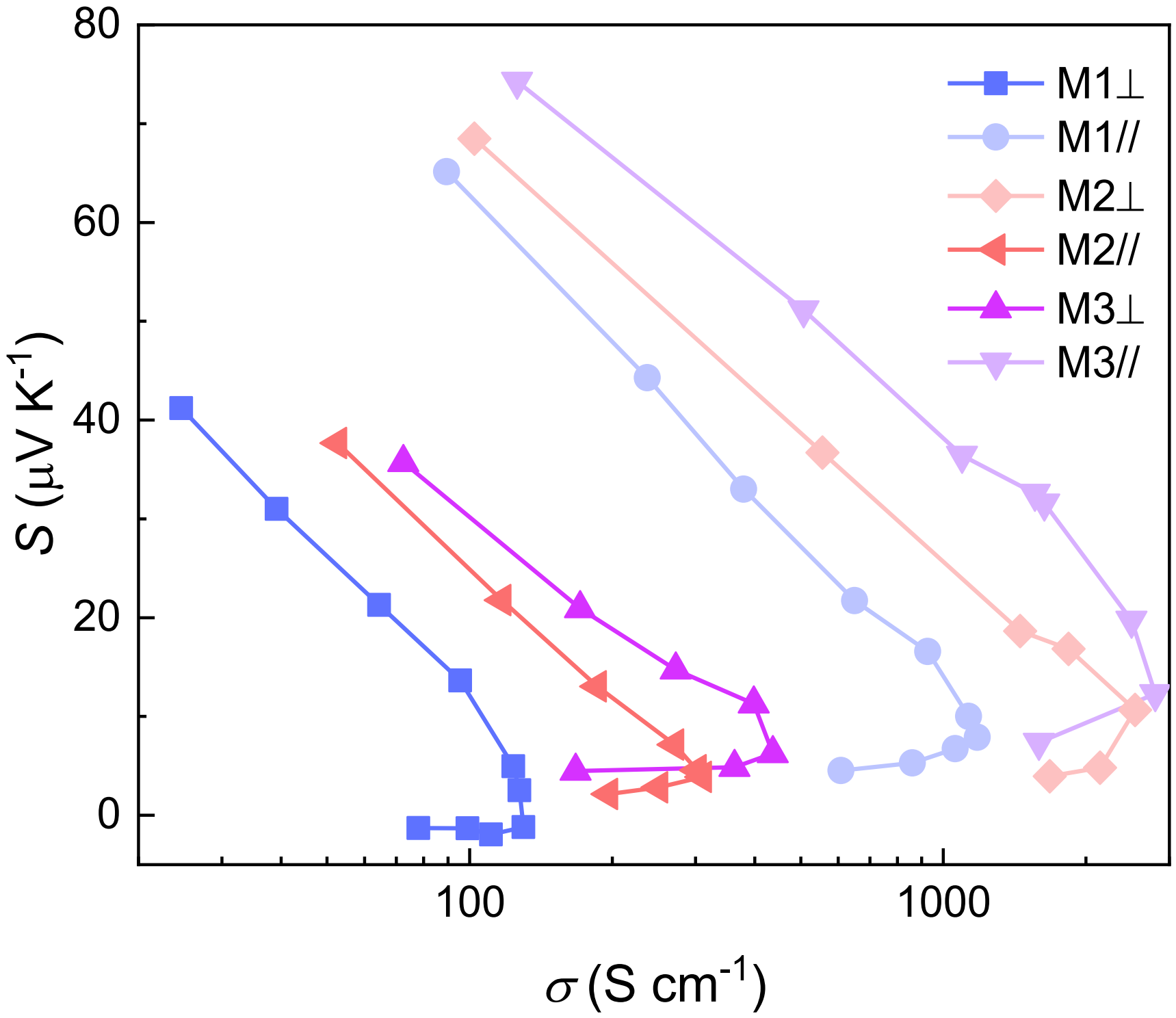

Fig. S12.

Effect of chain anisotropy in PBTTT thermopower and electrical conductivity with different molecular weight and alignment orientation. Specifically, the measured thermopower versus electrical conductivity in PBTTT $M_1^{\parallel}$, $M_1^{\perp}$, $M_2^{\parallel}$, $M_2^{\perp}$, $M_3^{\parallel}$, $M_3^{\perp}$ OECT devices with different gate voltages and doping level at 180 K are plotted here. The overall $S \sim \sigma$ trends are remarkably consistent across all molecular weights with a uniform slope prior to reaching maximum σ. In particular, perpendicular aligned PBTTT in general have much lower conductivity as compared to the parallel aligned devices. Also, larger molecular weight PBTTT with longer polymer chains have both larger Seebeck coefficients as well as conductivity than low molecular weight devices.

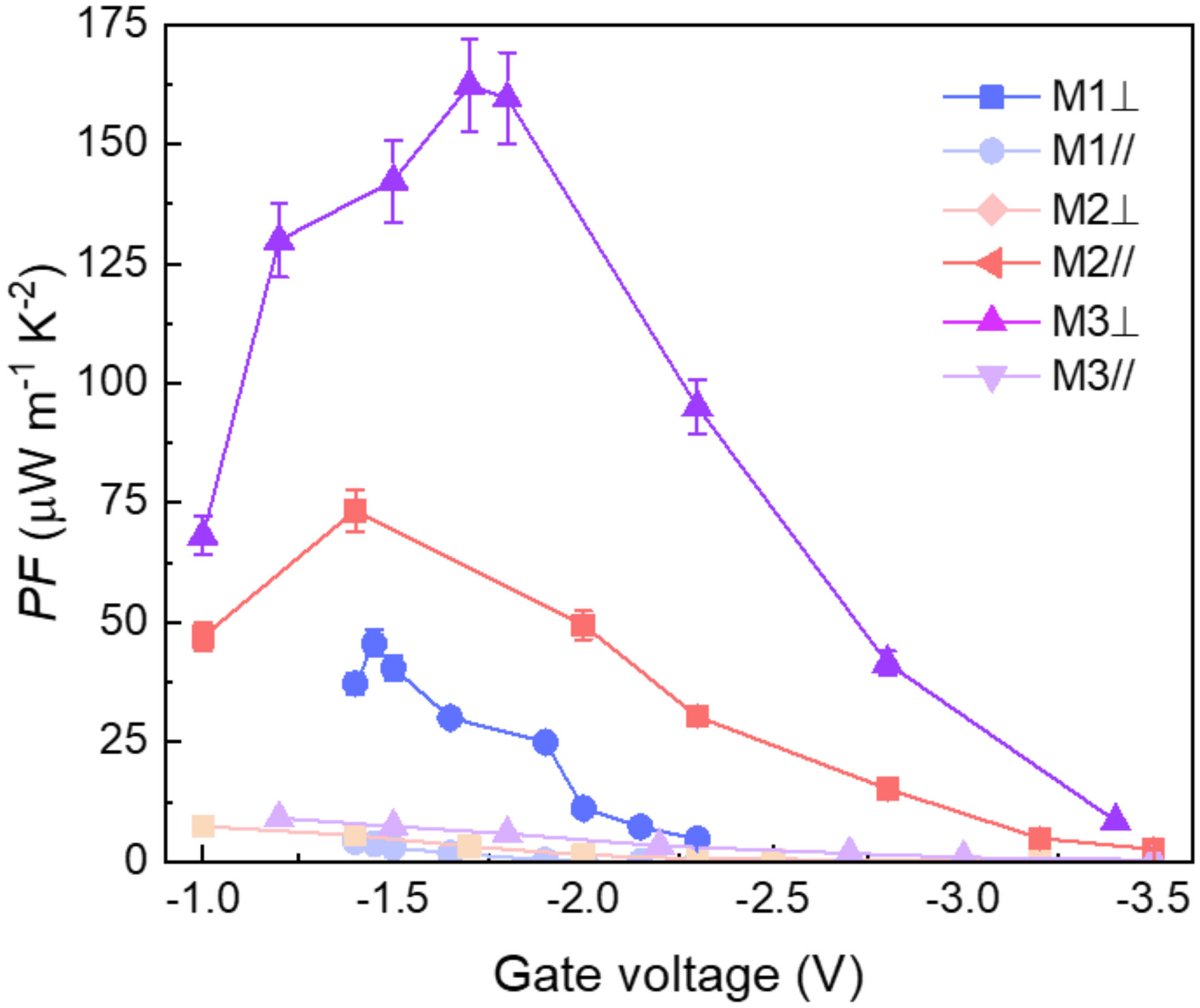

Fig. S13.

Plot of thermoelectric power factor (*PF*) as a function of gate voltage for PBTTT OECTs with varying molecular weights and alignments. A strong correlation is observed among molecular weight, anisotropy, and thermoelectric performance, with parallel-aligned or larger molecular weight devices consistently outperform perpendicularly aligned or lower molecular weight devices. This enhancement is attributed to the more efficient charge transport along the polymer backbone as compared to inter-chain hopping in aligned PBTTT films.

Supplementary Text

Supplementary Discussion 1:

Podzorov et al (*1*). proposed a model to explain the underdeveloped Hall voltage in organic transistors via a partial compensation effect between band-transport and hopping transport contributions. In this framework, band-transport (delocalized) carriers are deflected by the Lorentz force, generating the primary Hall voltage. Conversely, localized (hopping) carriers do not experience a classical Lorentz force; instead, they drift in response to the transverse electric field created by the band carriers, moving in the opposite direction. This counter-drifting hopping current acts to screen/cancel the net measured Hall voltage.

However, some highly conductive polymers (such as heavily doped PBTTT) exhibit (near-)metallic transport with nearly temperature-independent conductivities. The observation of non-activated conductivity indicates that the contribution from localized (hopping) carriers to the total conductivity is negligible, making the applicability of the Podzorov model to these heavily doped polymer systems questionable.

Supplementary Discussion 2:

In order to derive the expression of Hall coefficients in a quasi-1D (Q1D) system, we start with the general 2D tight-binding energy dispersion relation in the framework of semiclassical Boltzmann transport theory:

$$\epsilon\left(k_x, k_y\right) = -2t_x \cos(k_x a) - 2t_y \cos\left(k_y b\right) \tag{1}$$

in which t_x and t_y are transfer integral along and between polymer chains, respectively, and a and b are their corresponding lattice parameters. It should be noted that, in the Q1D transport limit, t_x should be much larger than t_y:

$$t_x \gg t_y \tag{2}$$

And the Fermi velocities are:

$$v_x = \frac{1}{\hbar}\frac{\partial E}{\partial k_x} \tag{3}$$

$$v_y = \frac{1}{\hbar}\frac{\partial E}{\partial k_y} \tag{4}$$

The longitudinal conductivity σ_{xx}, transverse conductivity σ_{yy}, and Hall conductivity σ_{xy} can then be derived from the velocities as integrals over the Fermi surface in the presence of magnetic field B_z:

$$\sigma_{xx} = \frac{q^2}{2\pi^2}\int \tau v_x^2 \left(-\frac{\partial f}{\partial E}\right) d^2k \tag{5}$$

$$\sigma_{yy} = \frac{q^2}{2\pi^2}\int \tau v_y^2 \left(-\frac{\partial f}{\partial E}\right) d^2k \tag{6}$$

$$\sigma_{xy} = \frac{q^3 B_z}{4\pi^2\hbar^2}\int \tau^2 \left(v_x^2 \frac{\partial^2 E}{\partial k_y^2} + v_y^2 \frac{\partial^2 E}{\partial k_x^2} - 2v_x v_y \frac{\partial^2 E}{\partial k_x \partial k_y}\right)\left(-\frac{\partial f}{\partial E}\right) d^2k \tag{7}$$

Specifically, for Q1D system ($t_x \gg t_y$), the Fermi surface consists of two nearly flat sheets at k_F. In this case, in the low magnetic field limit, the longitudinal conductivity σ_{xx}, transverse conductivity σ_{yy}, and Hall conductivity σ_{xy} can be expressed as:

$$\sigma_{xx} \approx \frac{4q^2\tau t_x a}{\pi\hbar^2 b}\sin(k_F a) \quad (8)$$

$$\sigma_{yy} \approx \frac{2q^2\tau t_y^2 b}{\pi\hbar^2 t_x a \sin(k_F a)} \quad (9)$$

$$\sigma_{xy} \approx \frac{4q^3\tau^2 t_y^2 abB_z}{\pi\hbar^4 \tan(k_F a)} \quad (10)$$

Where k_F is the Fermi vector:

$$k_F = \frac{\pi b}{2}n \quad (11)$$

Then the Hall coefficient R_H can be written as (*21*, *22*):

$$R_H = \frac{\sigma_{xy}}{\sigma_{xx}\sigma_{yy}B_z} = \frac{1}{nq}\frac{k_F a}{\tan(k_F a)} \quad (12)$$

Note that n represents the 2D charge density. It can be converted to 3D charge density by $n_{3D} = n/c$ (c is the lamellar spacing of the polymer). Physically, $\frac{k_F a}{\tan(k_F a)}$ can be considered as a filling factor. At low filling ($k_F a \sim 0$), the expression of R_H will reduce to the conventional formula: $R_H = \frac{1}{nq}$ and will be positive indicating p-type transport; at half-filling ($k_F a = \pi/2$), R_H will be zero indicating the transition point of p-type Hall to n-type Hall; over half-filling ($k_F a > \pi/2$), R_H will become negative, indicating n-type transport.

Supplementary Discussion 3:

The Q1D model is derived for systems exhibiting quasi-1D dispersion ($t_x \gg t_y$). We investigated whether this model can be extended to systems with lower levels of anisotropy. To address this, we performed an analysis based on a general 2D tight-binding model with different anisotropic transfer integrals. Unlike the Q1D model, the 2D model does not give a simple analytical expression and needs to be solved numerically. We calculated R_H as a function of the filling level for different ratios of t_y/t_x (1:5, 1:2 and 1:1). As shown in **Supplementary Figure 14**, the difference in R_H between the Q1D model and the 2D model is reasonably small in the heavily doped regime. An anisotropy ratio of 1:5 yields R_H values nearly identical to those of the Q1D model. Even in the isotropic case, for a given R_H, the difference in carrier density remains below 20%. Given that the conventional expression in literature usually leads to massive errors exceeding 1000%, the Q1D model provides a substantially more accurate way for carrier density extraction in heavily doped systems.

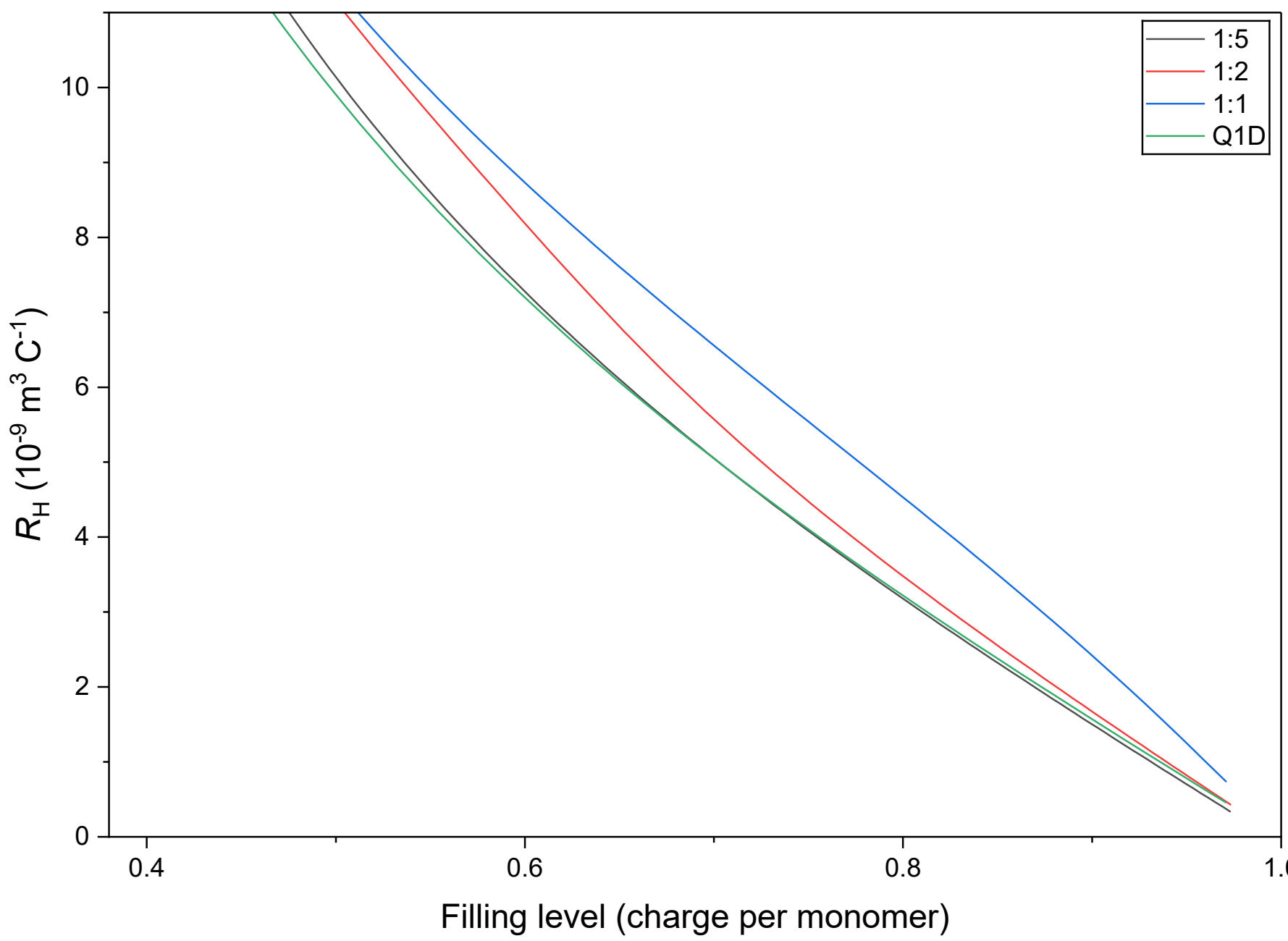

Fig. S14. Calculated hall coefficient as a function of filling level for different ratios of t_y/t_x.

The calculation of R_H assumes constant relaxation time τ. While this assumption gives a reasonably accurate estimation of R_H (**Supplementary Figure 15a**), its predictions for the Seebeck coefficients along the 2 directions are not fully consistent with experimental results. In the framework of semiclassical Boltzmann transport theory, the Seebeck coefficients (S_{xx} and S_{yy}) are given by:

$$S_{xx} = \frac{1}{eT}\frac{\int \tau v_x^2 (E - E_F)\left(-\frac{\partial f}{\partial E}\right) d^2k}{\int \tau v_x^2 \left(-\frac{\partial f}{\partial E}\right) d^2k} \tag{13}$$

$$S_{yy} = \frac{1}{eT}\frac{\int \tau v_y^2 (E - E_F)\left(-\frac{\partial f}{\partial E}\right) d^2k}{\int \tau v_y^2 \left(-\frac{\partial f}{\partial E}\right) d^2k} \tag{14}$$

If anisotropy exists (e.g., a ratio of 1:5), at filling levels of about 0.4 charge per monomer, S_{xx} and S_{yy} exhibit opposite signs. Experimentally, however, they have the same sign, although their magnitudes can be different. The sign of S is determined by the energy-dependent conductivity, and the negative sign of S_{yy} originates from the saddle points in the band structure. Under constant relaxation time approximation, σ roughly scales with $v^2 D(E)$. After the saddle point, while $D(E)$ dropping with increasing energy, the change of v^2 can be very different along the two directions: v^2 rises rapidly along the *x*-direction, while the increase along the *y*-direction is heavily suppressed due to the smaller transfer integral. Consequently, σ increases along the *x*-direction and decreases along the *y*-direction, leading to opposite signs in S near the saddle point.

To reconcile the calculated results with experimental observations, the energy dependence of τ must be considered. Previous literature indicates that the S-σ relationship in various polymers, including PBTTT, can be well described by an energy dependence of $\tau \propto E^{1.5}$, which is characteristic of an ionized impurities scattering mechanism (*27*). Incorporating this energy-dependent relaxation time makes the calculated S_{xx} and S_{yy} exhibit the same sign (**Supplementary Figure 15b**). Interestingly, this model also predicts $S_{yy} < S_{xx}$ at the same doping level, which is consistent with experimental observations in rub-aligned PBTTT (*28*).

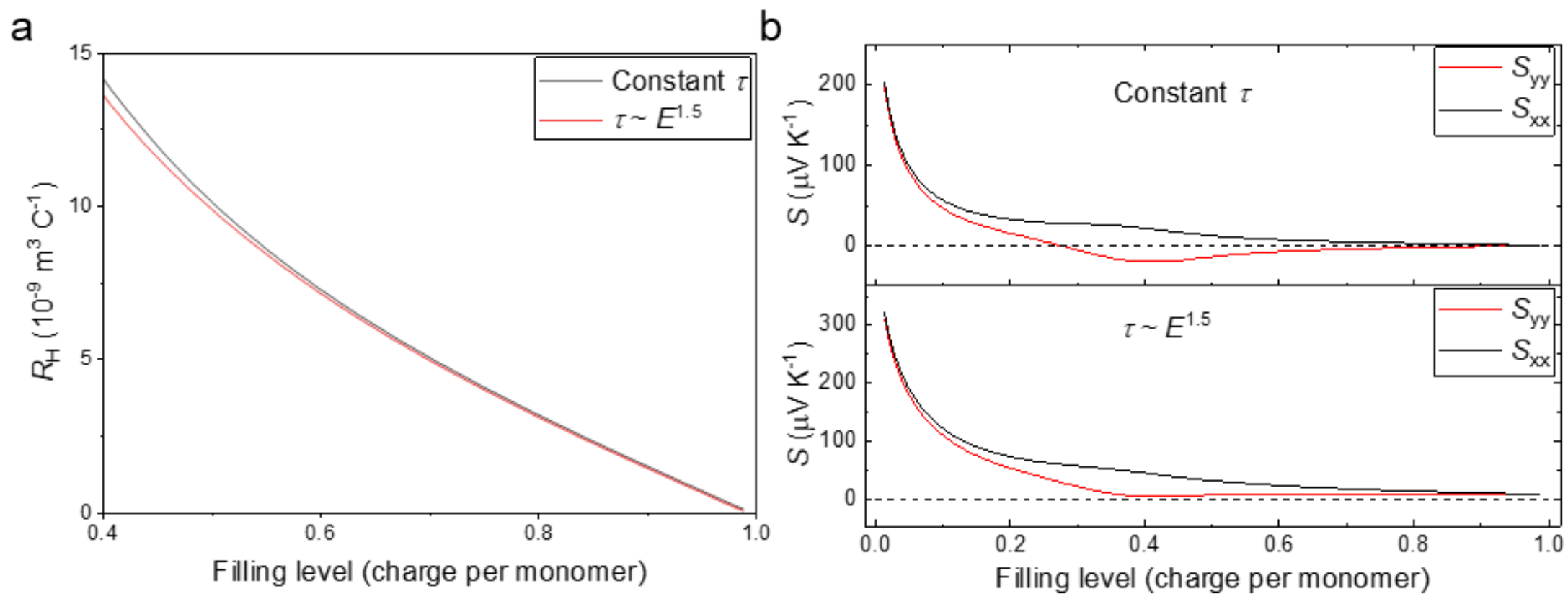

Fig. S15. Influence of energy-dependent relaxation time. (a) For heavily doped systems, the energy dependence of relaxation time has negligible impact on the Hall coefficient. (b) Constant τ leads to negative S_{yy} at ~0.4 charge per monomer, while S_{yy} remains positive for $\tau \propto E^{1.5}$. A transfer integral anisotropy of 1:5 is used in the calculations.

REFERENCES

1. H. Yi, Y. Gartstein, V. Podzorov, Charge carrier coherence and Hall effect in organic semiconductors. *Sci. Rep.* **6**, 23650 (2016).

2. Y. Yamashita, F. Hinkel, T. Marszalek, W. Zajaczkowski, W. Pisula, M. Baumgarten, H. Matsui, K. Müllen, J. Takeya, Mobility exceeding 10 $cm^2/(V\cdot s)$ in donor−acceptor polymer transistors with band-like charge transport. *Chem. Mater.* **28**, 420–424 (2016).

3. W. Wood, I. Jacobs, L. Spalek, Y. Huang, C. Chen, X. Ren, H. Sirringhaus, Revealing contributions to conduction from transport within ordered and disordered regions in highly doped conjugated polymers through analysis of temperature-dependent Hall measurements. *Phys. Rev. Mater.* **7**, 034603 (2023).

4. I. Jacobs, G. D'Avino, V. Lemaur, Y. Lin, Y. Huang, C. Chen, T. F. Harrelson, W. Wood, L. Spalek, T. Mustafa, C. O'Keefe, X. Ren, D. Simatos, D. Tjhe, M. Statz, J. Strzalka, J. Lee, I. McCulloch, S. Fratini, D. Beljonne, H. Sirringhaus, Structural and dynamic disorder, not ionic trapping, controls charge transport in highly doped conducting polymers. *J. Am. Chem. Soc.* **144**, 3005–3019 (2022).

5. Y. Ma, X. Ren, D. Wang, Y. Zou, W. Zhao, Z. Ji, J. Fan, C. Yan, L. Xiang, G. Ge, X. Dai, F. Zhang, T. Lei, H. Sirringhaus, C. Di, D. Zhu, Observation of anomalously large Nernst effects in conducting polymers. *Nat. Commun.* **16**, 1435 (2025).

6. H. Tang, Z. Xu, Y. Liang, W. Cui, Y. Chen, Q. Jiang, T. Lei, Y. Ma, F. Huang, Highly conductive alcohol-processable n-type conducting polymer enabled by finely tuned electrostatic interactions for green organic electronics. *Angew. Chem. Int. Ed.* **64**, e202415349 (2025).

7. W. Cui, Q. Jiang, X. He, S. Tong, B. Wang, M. Sun, H. Gan, Y. Ma, Charge transport Properties of PBFDO at various doping levels: An electrochemical control and hall effect characterization study. *J. Phys. Chem. Lett.* **16**, 6393–6401 (2025).

8. X. Zhang, J. Ding, D. Wang, X. Dai, Y. Ma, Y. Zhao, B. Guan, L. Liu, Y. Zou, F. Zhang, C. Di, Waterborne paints based on polymeric semiconductor for attachable thermoelectric generators. *Small Struct.* **4**, 2200278 (2023).

9. V. Podzorov, E. Menard, J. A. Rogers, M. E. Gershenson, Hall effect in the accumulation layers on the surface of organic semiconductors. *Phys. Rev. Lett.* **95**, 226601 (2005).

10. Y. Yamashita, J. Tsurumi, F. Hinkel, Y. Okada, J. Soeda, W. Zajaczkowski, M. Baumgarten, W. Pisula, H. Matsui, K. Müllen, J. Takeya, Transition between band and hopping transport in polymer field-effect transistors. *Adv. Mater.* **26**, 8169–8173 (2014).

11. S. P. Senanayak, A. Z. Ashar, C. Kanimozhi, S. Patil, K. S. Narayan, Room-temperature bandlike transport and Hall effect in a high-mobility ambipolar polymer. *Phys. Rev. B* **91**, 115302 (2015).

12. J. Chang, T. Sakanoue, Y. Olivier, T. Uemura, M. Dufourg-Madec, S. G. Yeates, J. Cornil, J. Takeya, A. Troisi, H. Sirringhaus, Hall-effect measurements probing the degree of charge-carrier delocalization in solution-processed crystalline molecular semiconductors. *Phys. Rev. Lett.* **107**, 066601 (2011).

13. Z. Liang, H. Choi, X. Luo, T. Liu, A. Abtahi, U. Ramasamy, J. Hitron, K. Baustert, J. Hempel, A. Boehm, A. Ansary, D. Strachan, J. Mei, C. Risko, V. Podzorov, K. Graham, n-type charge transport in heavily p-doped polymers. *Nat. Mater.* **20**, 518–524 (2021).

14. J. Liu, G. Ye, B. Zee, J. Dong, X. Qiu, Y. Liu, G. Portale, R. C. Chiechi, L. J. Koster, n-type organic thermoelectrics of donor–acceptor copolymers: Improved power factor by molecular tailoring of the density of states. *Adv. Mater.* **30**, e1804290 (2018).

15. Y. Ma, W. Zhao, D. Wang, X. Dai, Z. Ji, Z. Li, J. Ding, Z. He, L. Xiang, L. Liu, Y. Zou, C. Di, D. Zhu, Revealing unipolar thermoelectric performance in bipolar polymer. *Natl. Sci. Open* **4**, 20250001 (2025).

16. D. Tjhe, X. Ren, I. Jacobs, G. D'Avino , T. B. E. Mustafa, T. G. Marsh, L. Zhang, Y. Fu, A. E. Mansour, A. Opitz , Y. Huang, W. Zhu, A. Unal, S. Hoek, V. Lemaur , C. Quarti, Q. He, J. Lee,

I. McCulloch, M. Heeney, N. Koch, C. P. Grey, D. Beljonne, S. Fratini, H. Sirringhaus, Non-equilibrium transport in polymer mixed ionic–electronic conductors at ultrahigh charge densities. *Nat. Mater.* **23**, 1712–1719 (2024).

17. W. Zhu, X. Qiu, J. E. M. Laulainen, H. Un, X. Ren, M. Xiao, G. Freychet, P. Vacek, D. Tjhe, Q. He, W. Wood, Z. Wang, Y. Zhang, Z. Qu, J. Asatryan, J. Martin, M. Heeney, C. R. McNeill, P. A. Midgley, I. Jacobs, H. Sirringhaus, Enhancing the conductivity and thermoelectric performance of semicrystalline conducting polymers through controlled tie chain incorporation. *Adv. Mater.* **36**, 2310480 (2024).

18. N. Cusack, P. Kendall, The absolute scale of thermoelectric power at high temperatwe. *Proc. Phys. Soc.* **72**, 898–901 (1958).

19. R. B. Roberts, The absolute scale of thermoelectricity. *Philos. Mag.* **36**, 91–107 (1977).

20. J. E. Northrup, Atomic and electronic structure of polymer organic semiconductors: P3HT, PQT, and PBTTT. *Phy. Rev. B* **76**, 245202 (2007).

21. J. R. Cooper, M. Miljak, G. Delplanque, D. Jérome, M. Weger, J. M. Fabre, L. Giral, Dc Hall effect measurements on ttf-tcnq. *J. Phys. France* **38**, 1097–1103 (1977).

22. N. Wakeham, N. E. Hussey, Hall effect in quasi-one-dimensional metals in the presence of anisotropic scattering. *Phy. Rev. B* **85**, 235117 (2012).

23. I. McCulloch, M. Heeney, C. Bailey, K. Genevicius, I. MacDonald, M. Shkunov, D. Sparrowe, S. Tierney, R. Wagner, W. Zhang, M. L. Chabinyc, R. Kline, M. D. McGehee, M. F. Toney, Liquid-crystalline semiconducting polymers with high charge-carrier mobility. *Nat. Mater.* **5**, 328–333 (2006).

24. H. Tang, Y. Liang, C. Liu, Z. Hu, Y. Deng, H. Guo, Z. Yu, A. Song, H. Zhao, D. Zhao, Y. Zhang, X. Guo, J. Pei, Y. Ma, Y. Cao, F. Huang, A solution-processed n-type conducting polymer with ultrahigh conductivity. *Nature* **611**, 271–277 (2022).

25. C. Frisbie, I. Jacobs, X. Ren, H. Sirringhaus, Charge transport physics of organic conductors at high carrier densities. *Nat. Rev. Mater.* **11**, 437–452 (2026).

26. S. Kang, G. Snyder, Charge-transport model for conducting polymers. *Nat. Mater.* **16**, 252–257 (2017).

27. A. R. Chew, R. Ghosh, V. Pakhnyuk, J. Onorato, E. C. Davidson, R. A. Segalman, C. K. Luscombe, F. C. Spano, A. Salleo, Unraveling the effect of conformational and electronic disorder in the charge transport processes of semiconducting polymers. *Adv. Funct. Mater.* **28**, 1804142 (2018).

28. H. Zeng, P. Durand, S. Guchait, L. Herrmann, C. Kiefer, N. Leclerc, M. Brinkmann, Optimizing chain alignment and preserving the pristine structure of single-ether based PBTTT helps improve thermoelectric properties in sequentially doped thin films. *J. Mater. Chem. C* **10**, 15883–15896 (2022).
